# The use of ash at Late Lower Paleolithic Qesem Cave, Israel—An integrated study of use-wear and residue analysis

**DOI:** 10.1371/journal.pone.0237502

**Published:** 2020-09-21

**Authors:** C. Lemorini, E. Cristiani, S. Cesaro, F. Venditti, A. Zupancich, A. Gopher

**Affiliations:** 1 LTFAPA Laboratory, Department of Classics, Sapienza University of Rome, Italy; 2 DANTE Laboratory, Sapienza University of Rome, Italy; 3 Scientific Methodologies Applied to Cultural Heritage (SMATCH), Rome, Italy; 4 Institute of Archaeology, Tel Aviv University, Tel Aviv, Israel; Max Planck Institute for the Science of Human History, GERMANY

## Abstract

Employing an integrated approach to investigate the use of Late Lower Paleolithic flint tools found at the site of Qesem Cave (Israel), we revealed a particular trace pattern related to the employment of ashes at the site. Using a designated collection of replica items and combining use-wear and residue (morphological analysis, FTIR, SEM-EDX) analyses, we revealed the intentional use of ashes in preserving foods for delayed consumption as well as hide for delayed processing. Our interpretation, we believe is the most plausible one since we were able to delineate the specific use-wear fingerprints of the intentional use of ashes for such purposes, suggesting that our approach might be useful for the recognition of other similar functional-behavioral patterns. Lastly, in support of previous findings at Qesem Cave, our current findings present evidence for the processing of organic matters intentionally mixed with ash, leading us to suggest that the inhabitants of Qesem Cave were proficient not only in the habitual use of fire but also of its main by-product, ash. Hence, we call for a reassessment of the timeline currently assigned to hominins’ utilization of ash for storing and processing vegetal foods and hide.

## 1. Introduction

The adaptive role of fire in human evolution has inspired a rich archaeological debate focused on “when” and “where” fire had originated, as well as its benefits in facilitating light, heat, security and other aspects of human life [1–3]. The general use of fire is commonly dated back to the Early-Middle Pleistocene [4, 5] whereas the habitual use of fire seems, at large, to have emerged during the later Middle Pleistocene [6, 7]. Fire-technology, including fire-making and maintenance, has been documented in only a few Late Pleistocene contexts [8, 9], suggesting that prior to the Upper Palaeolithic [1], pyro-technology was infrequently used.

Fire’s dietary role is firmly documented as well as supported by present-day experimental work [2] and archaeological data [10–12]. The fact that fire served as a hub of social activities is also well-documented both in ethnographic studies [13 and references therein] and archaeological studies [10, 14–18], where detailed spatial distributions of material culture and faunal remains have provided fair reconstructions of human activities around hearths.

While ethnography provides a plethora of records on the direct use of fire, it also shows the use of its major by-products–smoke and ashes, which are widely documented in ethnographic contexts. For example, smoking techniques are often applied to food and hide [19–22] while ashes are employed in food roasting, preservation of edible matters, preservation of hide, hygiene treatment of dwellings aimed at keeping insects and parasites at bay [23–28] and more. Unlike the direct use of fire, these are harder to trace retrospectively, leading direct archaeological evidence for such use to be of ephemeral nature or altogether missing.

In this article, we investigate the possibility of identifying the use of ashes in food cooking and storage, as well as the treatment of hide, demonstrating that such an identification is indeed possible by means of an integrated approach comprising use-wear and residue analyses, controlled experiments, and a corroborating blind test. We show that the use of ash in these activities has led to the development of specific polish and striations morphology and distribution patterns that are distinguishable from those originating in the processing of ash free organic matter or the accidental presence of ash in the work-setting itself. We employed our analyses to flint tools from the (blade-dominated) Amudian and the (Quina and demi-Quina scraper-dominated) Yabrudian assemblages of the Late Lower Paleolithic site of Qesem Cave, dated to 420–200 kya. The results indicate that the detection of these specific use-wear patterns and supporting residues in these assemblages attests to manipulation technologies of food and possibly also other perishable materials, such as hide. The innovation in our study is thus twofold: first, that such use-wear features are indeed distinct and detectable, and second, that these technologies may have significantly influenced crucial adaptive elements in human evolution, enabling hominins to consume more digestible and high energy food and to plan longer term activities.

### 1.1. The site and the Acheulo-Yabrudian Cultural Complex (AYCC)

Qesem Cave is a Late Lower Paleolithic site located in Israel, some 12 km east of Tel Aviv’s Mediterranean coastline. It was discovered almost 20 years ago and excavated until 2016 on behalf of Tel Aviv University. A stratigraphic sequence of ca. 11 m was assigned in its entirety to the Acheulo-Yabrudian Cultural Complex (AYCC) of the Late Lower Paleolithic period, dated by Uranium Series, TL and ESR to ca. 420–200 kya [29–31]. The sequence boasts two AYCC industries: the blade dominated Amudian and Quina (as well as the demi-Quina) dominated Yabrudian [32–36].

The archaeological evidence from Qesem Cave indicates a series of novel human behaviours, especially when compared to the predating Lower Paleolithic Acheulian. This is evidenced by a plethora of lithic techno-typological innovations, among which are found large-scale systematic blade production, the production of Quina and demi-Quina scrapers, large-scale recycling of discarded flakes and blades as well as patinated items, and the recycling of bone fragments into retouchers, to name but a few such innovations [37–42]. Another outstanding innovation was the early habitual, use of fire at the site [37, 43]. A fireplace comprising a sequence of ash layers covering an area of approximately 4 m^2^ was exposed in the central part of the cave and dated to ca. 300 kyr [29]. In addition to its role as a central fireplace attracting various daily activities [10, 31, 43], the abundance of burnt animal remains both within and around the hearth [10] demonstrate the well-established and intensive processing and consumption of game animals.

## 2 Materials, hypothesis and methods

### 2.1. The archaeological material

The exceptionally well-preserved lithic industry at Qesem Cave, has allowed for past detailed use-wear investigations and the identification of various activities carried out with blades, Quina scrapers and the (small) products of recycled flakes and blades in various areas of the site in both Amudian and Yabrudian assemblages (S1 Table in S1 File) [38, 44–48]. Butchering and hide-working are among the most frequently represented activities evidenced at the site, with the exception of the hearth area. Here, a greater variety of activities emerged involving blades and, to a lesser degree, recycling products, as well as a broad range of substances including fresh and dry hide, wood, herbaceous plants and other types of plants as well as various plant organs, such as underground storage organs (USOs) (see S2 Table in S1 File, S1 Fig for the detail of the inferred data from use-wear in the different areas of the site) [45, 47–48].

**Fig 1:**
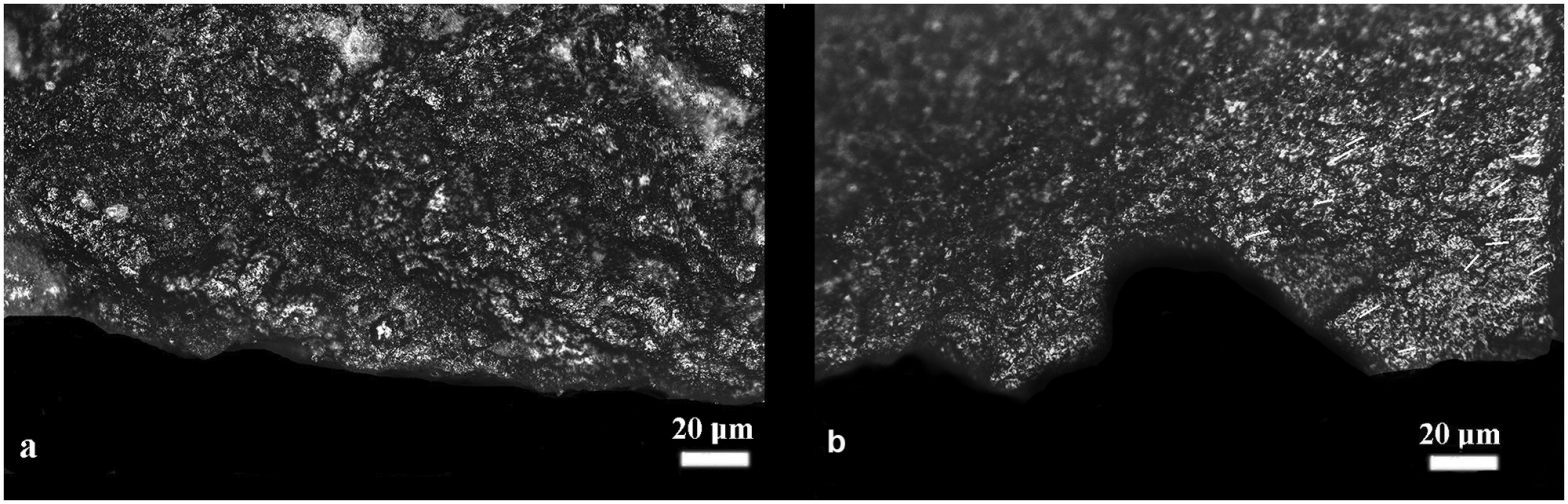
Examples of use-wear on archaeological lithic tools. a) use-wear (thin line of polish) of pure (ash-free) matters observed on item J13c 550–555 1 and of b) use-wear (band of bright polish and small thin striae highlighted by white lines) of pure matters + abrasive substance observed on item I12a 560–565 2.

Among the flint tools bearing use-wear marks (213 in total, see Table 1) a low percentage of flint tools (11%, n = 26; 20 blades, three small recycling products [flakes] and three scrapers, see Table 1), found mostly around the fireplace, exhibit use-wear with specific morphological features and distribution patterns on their active edges that were not observed on the other tools, meriting further examination. These features and patterns consist of a well-developed band of very bright polish, scratched by very fine striations and distributed along the active edge (Fig 1).

**Table 1:** Qesem Cave: a) specimen numbers of the lithic tools analysed and presented in this paper; b) Lithic items with use-wear from various areas of the cave.

**a)**					
	**Square**	**Elevations**	**N°**	**Area**	**Type**
**1**	I12a	560–565	2	Fireplace	Blade
**2**	I12b	560–565		Fireplace	Blade
**3**	I12c	560–565	2	Fireplace	Blade
**4**	I12c	575–580	2	Fireplace	Blade
**5**	I13a	560–565	1	Fireplace	Blade
**6**	I13a	575–580	3	Fireplace	Blade
**7**	I13b	580–585	5	Fireplace	Blade
**8**	I13b	590–595	1	Fireplace	Blade
**9**	I13b	590–595	3	Fireplace	Blade
**10**	I13c	580–585	2	Fireplace	Blade
**11**	I13c	580–585	3	Fireplace	Blade
**12**	I13d	585–590	1	Fireplace	Blade
**13**	I13d	590–595		Fireplace	Blade
**14**	I13d	690–695		Fireplace	Blade
**15**	J12a	560–565	1	Fireplace	Blade
**16**	J13a	600–605		Fireplace	Blade
**17**	J13c	550–555		Fireplace	Blade
**18**	J13c	585–595	2	Fireplace	Blade
**19**	J13c	595–600	1	Fireplace	Blade
**20**	J13d	595–600		Fireplace	Blade
**21**	I12b	560–565		Fireplace	Recycled Small Flake
**22**	I15d	585–590	2	South of Fireplace	Recycled Small Flake
**23**	J15a	585–590		South of Fireplace	Recycled Small Flake
**24**	D7b+d	1085–1095		Shelf	Quina Scraper
**25**	E11d	665–670		Shelf	Quina Scraper
**26**	E12b	580–595		Shelf	Quina Scraper
**b)**					
**Area of the Site**	**Categories of Tools Analyzed**	**No. of Items with Recognized Use-Wear**	**No. of Items with a New Specific Use-Wear Pattern**		
Fireplace	Blades (B), Quina (S) Scrapers, Recycled small flakes (R)	78	(B) 20+(R) 1 = 21		
Area South of the Fireplace	Recycled small flakes	10	2		
Square K10	Blades	74	-		
Shelf	Quina Scrapers	51	3		
**Total**		**213**	**26**		

The archaeological artefacts presented in this study are all stored at the Prehistoric Archaeology Laboratory at the Institute of Archaeology of the Tel Aviv University. No permits were required for the described study, which compiled with all relevant regulations.

### 2.2. Hypothesis

We thought that the brightness of the polish, and the tight linkage between polish and the striations suggest that animal and plant processing activities achieved with these tools involved some unknown abrasive powdery component that had the power to enhance the degree of levelling of the micro-surface of the used flint tools while also slightly grazing their surface. Because most (23 of 26) of these items (see Table 1) were found in the fireplace area and because ashes were found in site sediments [31], suggesting they were readily available at Qesem Cave, we hypothesized that ash could have caused this peculiar use-wear on the tools. This hypothesis was also supported by the strong similarity in morphology and use-wear distribution patterns found on the items studied and on items from hide-working experiments where hide was preserved in cold ashes before tanning that we carried out as part of our permanent reference collection at LTFAPA laboratory of Sapienza University.

Two facts seem to negate an argument in favor of (partly or wholly) unintentional use of ash or environmental or incidental contamination. The first is the fact that only 23 (20%) lithic tools (see Table 1) originating in the ash-rich area of the fireplace exhibit this peculiar use-wear. Should this use-wear pattern had been the result of environmental contamination, we would have expected it to have affected the majority, if not the entirety of lithic tools boasting use-wear that had originated in the vicinity of the fireplace. Second, the same peculiar use-wear patterns were observed on three flint tools retrieved from areas that were distant from the fireplace (see Table 1, the Shelf Area); that is, from archaeological layers in which ash was not as common as in the hearth area and the risk of a contamination was practically absent.

### 2.3. Methods

Evaluating the proposed hypothesis, we had to verify that the peculiar traces observed on the archaeological flint tools could indeed be related to an intentional, knowledge-based use of ashes aimed at manipulating organic matter, rather than to a haphazard contamination of the work-setting by environmental factors. To achieve this goal, we applied an integrated methodology that allowed us to investigate the issue through various perspectives. These were achieved using: (a) an experimental reference collection aimed at investigating in detail the characteristics of the residues and use-wear produced during the use of flint tools while intentionally working matters with ash; (b) a blind test verifying the distinct nature of these residues and use-wear compared to those developed in situations involving unintentional contact between ash, the worked matters, and the flint tools; (c) residue analyses of both the dedicated experimental series compared with the large reference collections available at DANTE and LTFAPA laboratories of Sapienza University and the archaeological items; (d) use-wear analyses of both the washed (see below) dedicated experimental series compared with the LTFAPA reference collection and the archaeological items. Information regarding all the equipment used in the following procedures is detailed in the SI.

#### 2.3.1. The dedicated experimental reference collection

**Fig 2:**
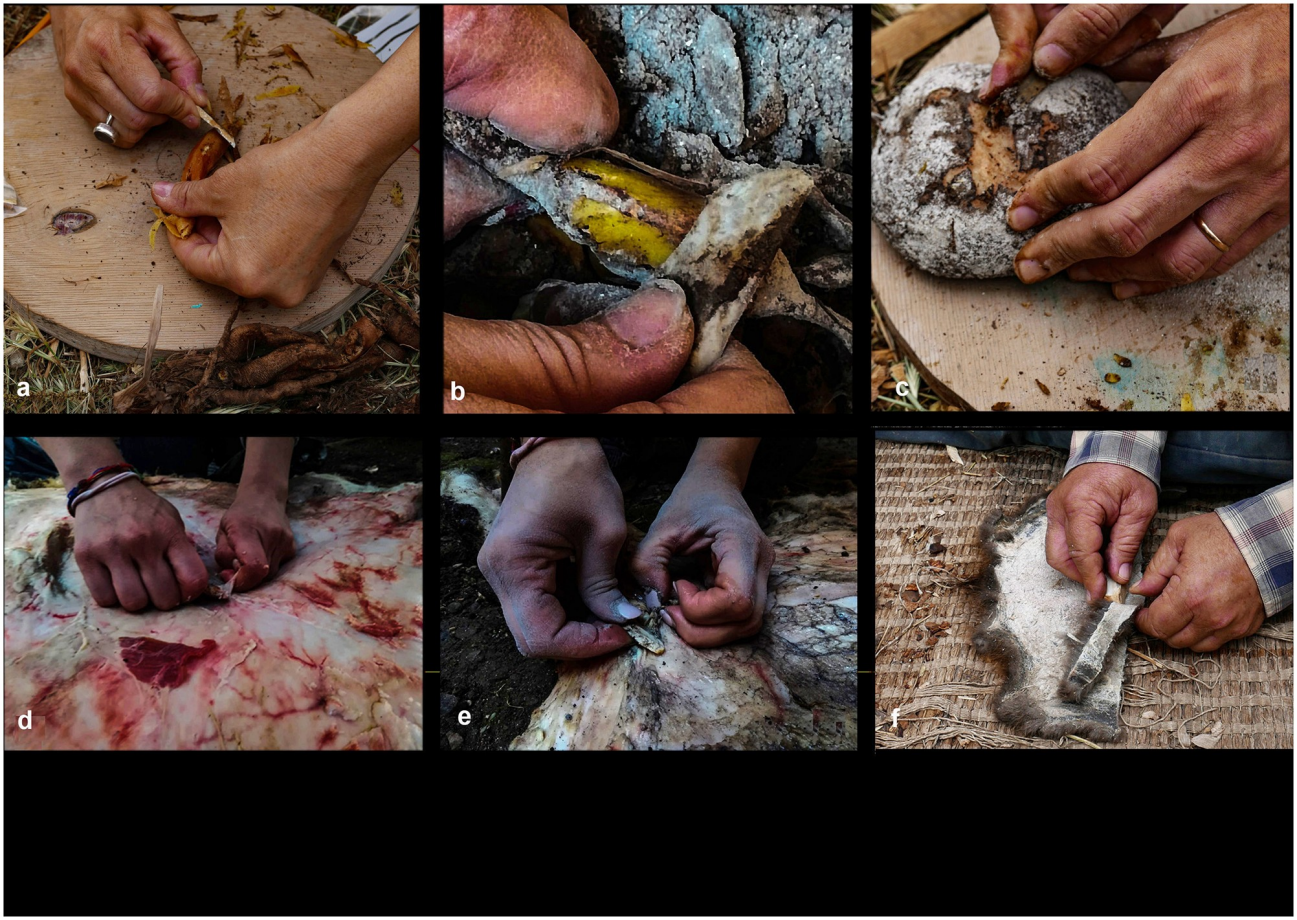
Experiments. a) peeling and cutting fresh asphodel; b) peeling and cutting fresh asphodel in ashy environment presence of ash (blind test); c) peeling roasted cyclamen; d) cleaning off subcutis of fresh hide (blind test); e) cleaning off subcutis of fresh hide in ashy environment (blind test; see also Repository Video 1); f) cutting dry hide preserved in ash.

In a series of 14 experiments (Table 2; Fig 2a, 2c and 2f), we created a dedicated reference collection) with which the archaeological artefacts were compared. In addition, the LTFAPA and DANTE laboratories large reference collection of experiments was available to us too. These collections document use-wear and residues of organic and inorganic matters including various matters preserved in ash for delayed processing such as hide and bone. The use of these robust collections and our own dedicated experiments allowed us to test the hypothesis that the traces observed on the archaeological items were related to the processing of ash-laden animal and plant matter (different types of USOs and herbaceous plant collectable in a Mediterranean environment similar to the Qesem Cave area) and to test the distinguishability of animal and plant matter stored and/or roasted in ash (ash-laden) and later worked by flint tools.

**Table 2:** List of the experiments that form the dedicated reference collection.

Exp. N°	Material Processed	Action	Working Time (min)
1	Fleshy tissues	Cutting	35
2	Fleshy tissues	Cutting	40
3	Fresh hide	Cutting (subcutis removal)	30
4	Fresh Cyclamen	Cutting	30
5	Fresh Cyclamen	Peeling	30
6	Fresh Leontis	Peeling	30
7	Fresh Leontis	Cutting	30
8	Fresh Asphodel	Peeling and cutting	110
9	Fresh wild chicory, wild fennel, burdock,	Cutting	60
10	Fresh dandelion, Mouritanicus grass, ivy	Cutting	60
11	Fresh Cyclamen, Asphodel, Leontis Preserved in Ash	Peeling and cutting	80
12	Dry Hide Preserved in Ash	Scraping and cutting	45
13	Roasted Cyclamen, Asphodel, Leontis	Cutting	56
14	Roasted Cyclamen, Asphodel, Leontis; Gundelia	Cutting	30

We used replicas of lithic tools in various experiments, each tool used for at least 30 minutes on various materials. The replicas were knapped from a flint source that is similar to the raw material from which the studied archaeological lithic items were made. Since various use wear analyses previously applied to lithic assemblages of Qesem Cave [38, 44–48] did not reveal consistent data supporting their hafting, blade replicas were all used free-hand. Experimental activities included ash-free cutting of fresh fleshy tissues, cutting fresh hide, scraping fresh hide, cutting dry hide, and scraping dry hide. Traditional recipes [19] recommend using salt or ash in order to prevent the decay of fresh hide when cleaning procedures are not practiced immediately after skinning. Fresh hide was left for several days covered by ash, dried, softened by scraping (Fig 2f), and, finally, cut into strips.

For the experimental processing of USOs, we chose *Asphodelus ramosus L*., *Leontice leontopetalum L*., *Cyclamen persicum Mill*., and *Gundelia tournefortii L*. due to their easy accessibility in the Mediterranean area and the traditional use of their sprouts and USOs for dietary purposes [49, 50]. Experimental activities involving USOs included peeling and slicing USOs when fresh (Fig 2a), peeling and slicing after preservation treatment under cold ashes for ten days, and peeling and slicing after roasting under ash (Fig 2c). In our experiments, hot ash was used for roasting USOs while cold ash was used for preserving whole raw USOs in an insect-repellent environment based on traditional food preservation practices prevalent in Africa and India [23, 25, 26, 28].

We additionally used replicas of flint tools for gathering edible herbaceous plants (wild chicory, wild tennel, burdock, dandelion) and for gathering herbaceous plants useful for basketry (Mouritanicus grass), since use-wear related to herbaceous plants, although rare, was found present among the Qesem Cave flint tools.

#### 2.3.2. Blind test

Another group of experiments, composed by eight flint replicas, was carried out by experimenters not directly involved in our analyses with the aim to create a sample of tools to be used for a blind test (Table 3). The eight flint replicas were used in activities involving: (a) ash-free matters, (b) matters intentionally mixed with ash, and (c) matters worked in ashy environment. Specifically, they were used in the processing of fresh ash-free hide (*n* = 1) (Fig 2d), fresh hide worked in an ashy environment (*n* = 1) (Fig 2e and Repository Video), raw hide preserved for a week in cold ash (*n* = 1), fresh ash-free USO (*n* = 1), fresh USOs worked in an ashy environment (*n* = 2) (Fig 2b), raw USO preserved for a week in cold ash (*n* = 1) and roasted USO (*n* = 1). These flint replicas were then analyzed by the present authors in order to 228 test the reliability of the identification of residues and the use-wear traces originating in ash 229 laden (the intentional use of ash) as opposed to ashy (accidental ash contamination due to the 230 ashy nature of the work-setting).

**Table 3:** List of the experiments that form the blind test.

Exp. N°	Material Processed	Action	Working Time
1	Fresh Hide	Cutting (Cleaning off subcutis)	1h
2	Fresh Hide presence of Ash	Cutting (Cleaning off subcutis)	1h
3	Fresh Asphodel	Peeling + Cutting	1h
4	Fresh Cyclamen, Asphodel, Gundelia, Leonitis preserved in Ash	Cutting	1h
5	Dry Hide preserved in Ash	Cutting + Scraping	1h5m
6	Fresh Asphodel presence of ash	Peeling + Cutting	1h20m
7	Fresh Asphodel presence of Ash	Peeling + Cutting	1h
8	Roasted Cyclamen, Asphodel, Leontis; Gundelia	Peeling	30m

#### 2.3.3. Washing procedure and manipulation of the lithic tools

All items were manipulated at all times with powder-free gloves. Once residues were analyzed, 236 both archeological items and replicas were washed in preparation for the use-wear analysis. The archeological items were initially washed with tap water, and subsequently treated in a bath of de-mineralized water with a 2% solution of buffered soap Derquim^©^ [51]. The items were then treated in an ultrasonic tank for 5 minutes, followed by a final rinsing using demineralized water in an ultrasonic tank for 5 minutes. The lithic replicas were initially washed following the same procedure that was applied to the archaeological items. However, it was found that this procedure was too gentle to eliminate all residues accumulated on these items. The replicas were thus first washed with running water and liquid soap after which they were left in diluted 3% acetic acid (CH3COOH) for 15 minutes followed by treatment in a diluted 3% sodium hydroxide (NaOH) base for additional 15 minutes. The items were then rinsed with de-mineralized water in an ultrasonic tank and further treated with the same washing procedure as the archeological tools.

#### 2.3.4. Residue analyses

Eight blades (31%) of the 26 items analyzed in this study were selected for testing both the possible presence of residues as well as the relevance of such residues for interpreting the specific features noted on the tools. Three independent methods were combined in our residue analyses: a morphological residue approach (EC and AZ) [52–54], Fourier Transform Infrared (FTIR) microspectroscopy (SNC) [55, 56], and SEM-EDX (CL) [57].

The nature of archaeological residues was interpreted based on their morpho-qualitative features (color, appearance, inclusions, consistency, biorefrengency, etc.), spatial patterns of distribution [53, 58], chemical characterization (in the current study, through two independent techniques: FTIR and SEM-EDX), and comparison with available literature discussing archaeological and experimental residues found on stone tools [57, 59–69]. We additionally compared the archaeological residues with two independent databases. One is the LTFAPA and DANTE laboratories’ collections of experimental residues produced while using flint implements in working bone, antler, natural and ochre-stained hide, tendons, wood, bark, siliceous plants, and adhesive hafting compounds (e.g., beeswax, resin, bitumen, animal bonebased glues). The other is our own dedicated collection of experimental implements (see Section 2.3.1 above) that were used in working fresh fleshy tissues, fresh hide, dry hide, herbaceous plants, ash-free USOs, and USOs mixed with ashes for roasting or preservation purposes.

#### 2.3.4. Use-wear analyses

Use-wear analysis, combining low- and high-power approaches [70–75], was performed by CL, FV and AZ on all 26 items analyzed in this study. The low-power approach is useful for obtaining a broad view of the localization of the use-traces on the tool, and it is useful for observing and describing edge-rounding and edge removals. Edge removals are represented by scars on the active edge created during use. These are defined based on the morphology of their fracture initiation and termination [76] along with their distribution and orientation. Edge rounding can be visible to a high or low degree or be indiscernible.

The high-power approach is useful for observing microwear, such as polish and striation along with micro-rounding of the edge. Polishes are described and interpreted based on their texture and topography along with their orientation and distribution. Striations are defined based on their morphology (depth, width and cross-section), orientation, and location. Micro rounding can be present to a high or low degree or be absent.

#### 2.3.5. Matching residues and use-wear

Observed residues were related to use by integrating the results of the use-wear study and the final interpretation of the residues. This allowed us to associate ancient residues with observed use while excluding the possibility of environmental or incidental contamination.

## 3. Results

### 3.1. Morphological residues blind test

In line with our assumption, both analysts (EC, AZ) in our blind test correctly identified the presence and absence of ash on all eight replicas analyzed at this phase (Table 4). The goal of the test, however, was to evaluate whether the intentional addition of ash to the processed matter could be identified based on specific patterns of residue morphology and distribution.

**Table 4:** Blind test results.

		Use-Wear Analysis			Residues Analysis		
N°exp	Matter s Worke d	Analyst #1 Active Edge	Analyst #2 Active Edge	Analyst #1 Prehensive Area	Analyst #2 Prehensive Area	Analyst #1	Analyst #2
1	Fresh hide	Matter not mixed with ash: correct Matter: indetermi nable	Matter not mixed with ash: correct Matter: correct	Presence/Ab sence of ash: correct	Presence/Ab sence of ash: correct	Matter: correct Presence/Absence of ash: correct Residue Distribution: Inner Edge	Matter: correct Presence/ Absence of ash: correct Residue Distribution: Inner Edge
2	Fresh hide presence of ash	Matter not mixed with ash: correct Matter: correct	Matter not mixed with ash: correct Matter: correct	Presence/ Absence of ash: correct	Presence/ Absence of ash: correct	Matter: correct Presence/ Absence of ash: correct Residue Distribution: Inner Edge	Matter: correct Presence/ Absence of ash: correct Residue Distribution: Inner Edge
3	Fresh USOs (aspho del)	Matter not mixed with ash: correct Matter: indetermi nable	Matter not mixed with ash: correct Matter: correct	Presence/Ab sence of Ash: correct	Presence/Ab sence of ash: correct	Matter: correct Presence/Ab sence of ash: correct Residue Distribution: Edge Extremities	Matter: correct Presence/Ab sence of ash: correct Residue Distribution: Edge Extremities
4	USOs (asphodel,cyclamen,leontis,gundelia) preserved in ash	Matter mixed with ash: correct Matter: correct	Matter mixed with ash: correct Matter: indeterminable	Presence/ Absence of ash: correct	Presence/ Absence of ash: correct	Matter: correct Presence/Ab sence of ash: correct Residue Distribution: Edge Extremities	Matter: correct Presence/Ab sence of ash: correct Residue Distribution: Edge Extremities
5	Hide preser ved in ash	Matter mixed with ash: correct Matter: correct	Matter mixed with ash: correct Matter: incorrect	Presence/ Absence of ash: correct	Presence/Ab sence of ash: correct	Matter: correct Presence/ Absence of ash: correct Residue Distribution: Inner Edge	Matter: correct Presence/ Absence of ash: correct Residue Distribution: Inner Edge
6	Fresh USOs (asphodel)Presence of ash	Matter not mixed with ash: correct	Matter not mixed with ash: correct	Presence/Ab sence of ash: correct	Presence/Ab sence of ash: correct	Matter: correct	Matter: correct
		Matter: correct	Matter: correct			Presence/ Absence of ash: correct Residue Distribution: Edge Extremities	Presence/ Absence of ash: correct Residue Distribution: Edge Extremities
7	Fresh USOs (asphodel)presence of ash	Matter not mixed with ash: incorrect Matter: correct	Matter not mixed correct Matter: correct	Presence of ash: correct	Presence of ash: correct	Matter: correct Presence/ Absence of ash: correct Residue Distribution: Edge Extremities	Matter: correct Presence/ Absence of ash: correct Residue Distribution: Edge Extremities
8	Roasted USOs (asphodel,cyclamen,leontis, gundelia)	Matter mixed with ash: correct Matter: correct	Matter mixed with ash: correct Matter: correct	Presence/Ab sence of ash: correct	Presence/Ab sence of ash: correct	Matter: incorrect Presence/ Absence of ash: correct Residue Distribution: Inner Edge	Matter: incorrect Presence/ Absence of ash: correct Residue Distribution: Inner Edge

Residues morphology and distribution were indeed informative vis-à-vis the activities–cutting or scraping–performed on different animal and vegetal materials leading our blinded analysts to correctly identify the nature and the condition (dry/fresh) of the processed matters. Residue morphology and distribution were found insufficient to define whether ash was intentionally added to or accidentally present in the activity, since in both cases the additive ash was mixed with the processed material. Not only the active edges, but the prehension (grip) areas, too, showed ash distribution as patches of mud-cracked, compressed ashes mixed with organic matter regardless of the actual activity (e.g., peeling roasted matters, working substances preserved under cold ashes, or working substances in proximity to a fireplace) (S2 Fig).

### 3.2. Micro-residues analysis

#### 3.2.1. Morphological analysis

The appearance and distribution of organic micro-residues on the studied sample of archaeological blades, as observed under both low and high magnification, indicate activities involving animal and plant matters (Tables 5 and 6).

**Table 5:** Comparative table of residues and use-wear analyses of the active edge of the archeological lithic items.

Artefact Id	Residues Description Active Edge	FTIR Active Edge	SEM EDX Active Edge	Use-Wear Active Edge	Interpretation
**I13a 560–565 1**	Amorphous accumulation of whitish translucent greasy residues away from the edge. Yellowish, glossy mud-cracked residue			**Edge Removals:** Feather/Step **Edge-Rounding:** Yes **Polish Texture:** Rough to Smooth and Smooth **Striae:**Narrow/Shallow/Short;Oblique	**Residues:** Animal Fat **Use-Wear:** Soft Material + Ash; Cutting
**13b 580–585 5**	Amorphous accumulation of whitish translucent greasy residues along the edge		Carbon Oxygen Silicon Calcium Aluminum	**Edge Removals:** Feather/Snap/Step;Oblique **Edge- Rounding:** Yes **Polish Texture:** Smooth **Striae**: Narrow/ Shallow/Long **Polish Topography:** Granular to Flat	**Residues:** Animal Fat SEM-EDX: Organic Material, Flint, Ash, Soil **Use-Wear**: Dry Hide + Ash, Cutting
**I13c 580–585 3**	Amorphous accumulation of greasy whitish translucent residues, along and far from the edge	2913 w C-H st* 2844 w C-H st* 1571 w C-O st* 1536 w C-O st*		**Edge-Removals:** Feather/Hinge **Edge-Rounding:** Yes **Polish Texture:** Smooth **Striae:**Narrow/Shallow/Short; Oblique	**Residues:** Animal Fat FTIR: Fat Acid Salt **Use-Wear:** Semi-Dry Hide + Ash; Cutting
				**Polish Topography:** Flat	
**I13d 585–590 1**	Accumulations of whitish-beige residues with a fibrous structure, all over the tool, compressed appearance is consistent with prehension. One white plant fibre	2913 w C-H st* 2844 w C-H st* 1571 w C-O st* 1536 w C-O st* ~913 sh+ ~1450 vw, br§ 877 vw§	Oxygen Carbon Silicon Calcium Aluminum	**Edge-Removals:** Feather/Step; Oblique **Edge-Rounding:** Yes Polish Texture: Smooth and Rough to Smooth **Striae:** Narrow/Shallow/Short **Polish Topography:** Domed with Flat Spots	**Residues:** Plant tissues FTIR: *Fat Acid Salt; + Kaolin (Soil); §Ash SEM-EDX: Organic Material, Flint, Ash, Soil **Use-Wear:** USOs +Ash; Cutting
**I13d 690–695**	No residues identified			**Edge-Removals:** Feather/Hinge/Step; Oblique **Edge-Rounding:** Yes **Polish Texture:** Smooth + **Striae:** Narrow/Shallow/Short **Polish Topography:** Domed	**Use-Wear:** Semi-Dry Hide + Ash; Cutting
**I13b 590–595 1**	Amorphous accumulation of whitish greasy residue along the edge Patches of whitish and brownish residues mixed with plant fibres along the edge	~913 sh	Oxygen Silicon Carbon Aluminum Iron Calcium Magnesium	**Edge-Removals:** Feather **Edge-Rounding:** Yes **Polish Texture:** Rough to Smooth **Striae:**Narrow/Shallow/Short; Oblique **Polish Topography:** Granular to Domed	**Residues:** Animal Fat + Plant Tissues FTIR: Kaolin(Soil) SEM-EDX: Flint, Organic Material, Soil, Ash **Use-Wear:** USOs +Ash; Cutting
**I13b 590–595 3**	Yellowish residues with liquid/sticky appearance, patchy distribution, clear directionality and “mudcracked appearance”. Black residues consistent with soil		Carbon Oxygen Silicon Calcium Aluminum	**Edge-Removals:** Feather/Step; Oblique/ Perpendicular **Polish Texture:** Smooth + Few**Striae:** Narrow/Shallow/Short **Polish Topography:** Flat	**Residues:** Plant tissues with presence of Soil particles SEM-EDX: Organic Material, Ash, Soil **Use-Wear:** Herbaceous plant + Ash; Mixed Motion
**I13d 590–595 1**	Amorphous accumulations of whitish greasy residues, distributed along the edge. Collagen fibre	2913 w C-H st 2844 w C-H st 1571 w C-O st 1536 w C-O st	Oxygen Carbon Silicon Calcium Iron Aluminum	**Edge-Removals:** Feather/Step **Edge-Rounding:** Yes Polish Texture: Rough to Smooth **Striae:** Wide/ Deep/Long; Oblique **Polish topography:** Granular with Flat Spots	**Residues:** Animal Fat and Fibres FTIR: Fat Acid Salt SEM-EDX: Organic Material, Flint, Ash, Soil **Use-Wear:** USOs + Ash; Cutting

**Table 6:** Comparative table of residues and use-wear analyses of the prehension area of the archeological lithic items.

Artefact Id	Residues Description Prehension Area	FTIR Active Edge	SEM EDX Prehension Area	Use-Wear Prehension Area	Interpretation
**I13a 560–565 1**		2913 w C-H st°* 1647 w, br° 1571 w C-O st* 1535 w C-O st* ~1450 vw, br§ 877 sh§		**Edge-Rounding:** Yes (Cortex) **Polish Texture:** Smooth **Striae:**Narrow/Shallow **Polish Topography:** Domed	**Residues:** FTIR: *Fat acid salt, °Proteins, §Ash **Use-Wear:** Free-hand Prehension + Ash
**13b 580–585 5**		2913 w C-H st 2844 w C-H st 1571 w C-O st 1536 w C-O st		**Edge-Rounding:** Yes **Edge-Removals:** Hinge **Polish Texture:** Smooth **Striae:** Narrow/Shallow **Polish Topography:** Granular	**Residues:** FTIR: Fat acid salt **Use-Wear:** Free-hand Prehension + Ash
**I13c 580–585 3**		2913 w C-H st * 2844 w C-H st * 1571 w C-O st* 1536 w C-O st*		**Edge-Removals:** Feather **Edge-Rounding:** Yes	**Residues:**FTIR: *Fat acid salt, +Kaolin (Soil) **Use-Wear:** Free-hand Prehension + Ash
		~913 sh+		**Polish Texture:** Smooth **Striae:** Narrow/Shallow **Polish Topography:** Domed	
**I13d 585–590 1**				**Edge-Removals:** Feather **Edge-Rounding:** Yes	**Use-Wear:** Free-hand Prehension
**I13d 690–695**		~913 sh+ ~1450 mw, br§ 877 w§		**Edge-Removals:** Feather **Edge-Rounding:** Yes Polish Texture: Smooth **Striae:** Narrow/Shallow **Polish Topography:** Domed	**Residues:**FTIR: +Kaolin (Soil), §Ash **Use-Wear:** Free-hand Prehension
**I13b 590–595 1**				**Polish Texture:** Smooth **Straie:** Narrow/Shallow **Polish Topography:** Reticulated/Domed	**Use-Wear:** Probably Free-hand Prehension + Ash
**I13b 590–595 3**		2913 w C-H st* 2844 w C-H st* 1571 w C-O st* 1536 w C-O st* ~913 sh+		**Edge-Rounding:** Yes	**Residues:**FTIR: *Fat acid salt, +Kaolin (Soil) **Use-Wear:** Probably Free-hand Prehension
**I13d 590–595 1**		2913 w C-H st* 2844 w C-H st* 1571 w C-O st* 1536 w C-O st* ~913 sh+ ~ 1450 vw, br§ 877 sh§	Oxygene Carbon Silicon Calcium Iron Aluminium	Edge-Rounding: Yes (Cortex) **Polish Texture:** Smooth **Striae:** Narrow/Shallow **Polish Topography:** Domed	**Residues:**FTIR: *Fat acid salt, +Kaolin (Soil), §Ash SEM-EDX: Organic material, Flint, Soil, Ash **Use-Wear:** Free-hand Prehension + Ash

**Fig 3:**
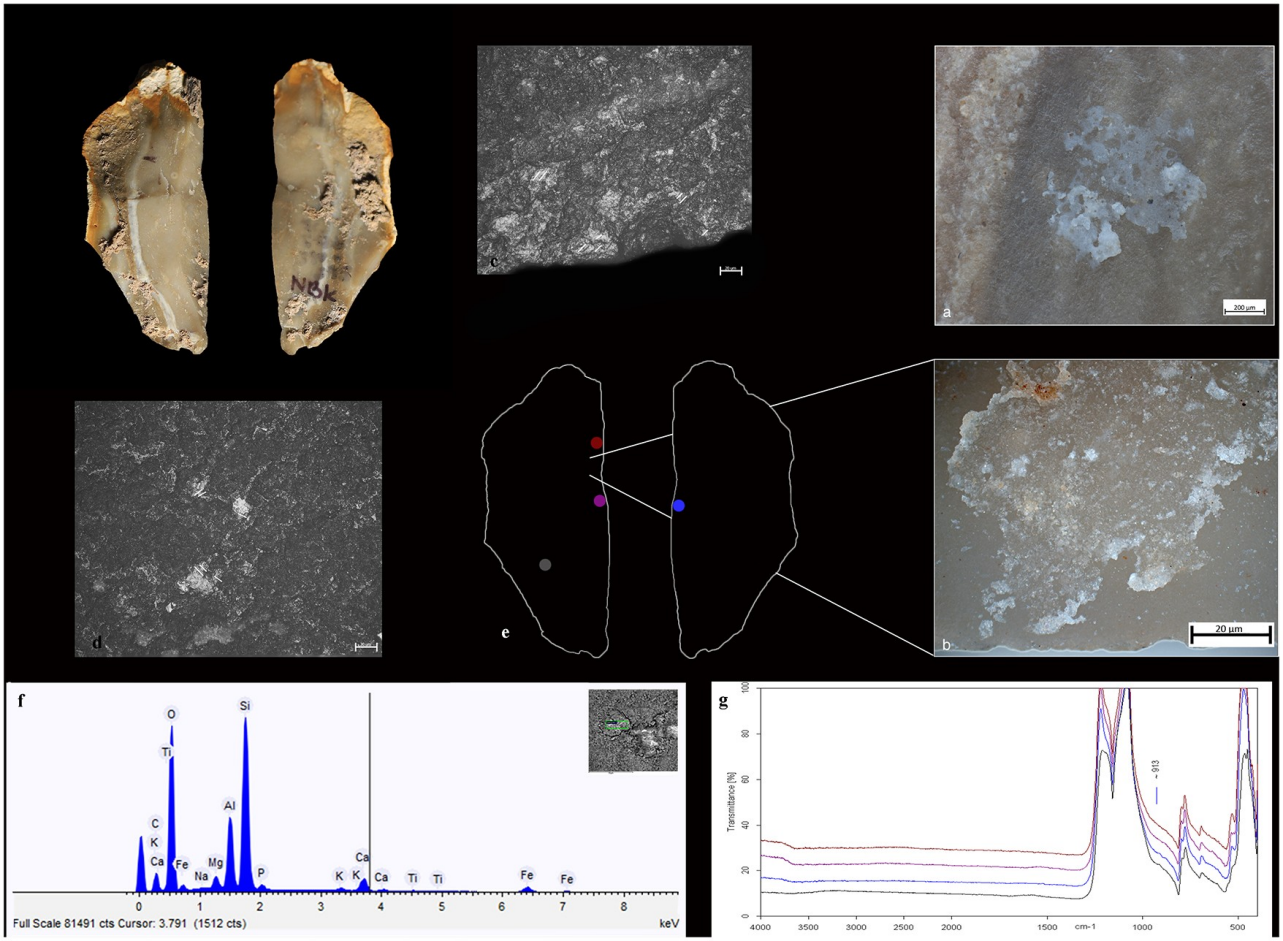
Qesem Cave, item I13b 590–595 1: a) experimental residue remains following the cutting and scraping of ash-laden fresh USOs; b) archeological residue on artefact I13b 590-595-1; c) band of use-wear resulting from contact with USOs; d) spots of polish resulting from free-hand prehension and contact with ash; e) item delineation showing the areas of FTIR analysis (in color) and areas of macro-residues; f) SEM-EDX spectrum of residues on the active edge; g) MicroFTIR spectrum of the ventral active area (blue).

**Fig 4:**
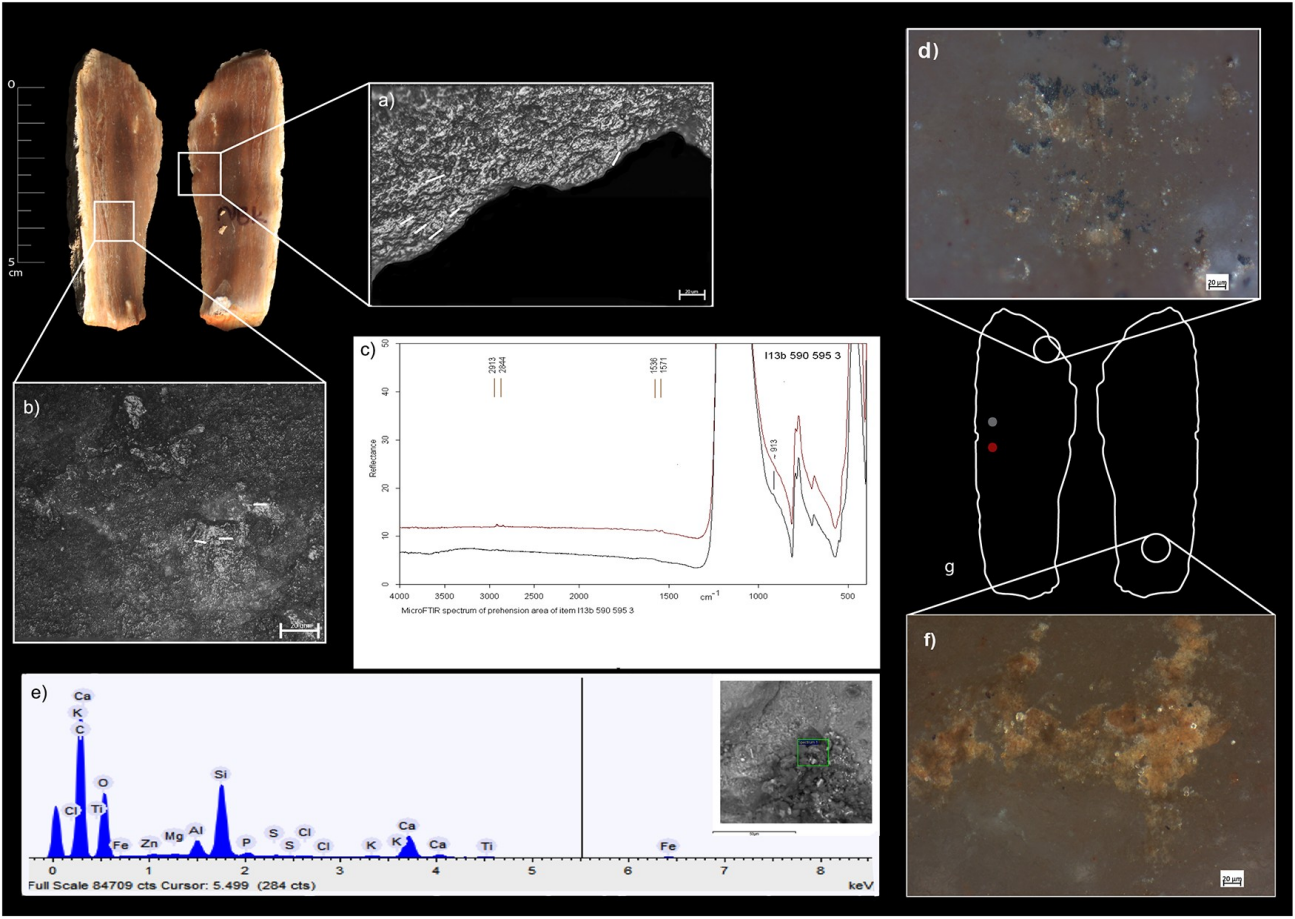
Qesem Cave, item I13b 590–595 3: a) use-wear resulting from contact with ash-laden herbaceous plant; b) spot of use-wear resulting from free-hand prehension and contact with ash; c) MicroFTIR spectrum of prehension area; d) archaeological residues consistent with soil and plant structures; e) SEM-EDX spectrum of residues on the active edge; f) archaeological organic film and an accumulation of yellowish residue consistent with plant material; g) item delineation showing the areas of FTIR analysis (in red) and areas of macro-residues.

Animal residual material is consistent with fat substances, collagen, and muscle fibers associated with butchering while plant residues comprise organic films, amorphous fiber-rich structures (Figs 3a, 3b, 3e and 4d, 4f and 4g), and soil particles. On the active edges of the tools, plant residues exhibit an invasive and patchy distribution. These residues are associated with plant-related polish in patterns that have been experimentally observed using flint replicas in processing energy-rich plants such as USOs (S3 Fig). The archaeological plant residues, however, may not be attributed to any specific plant species based on their dimensions and morphology.

#### 3.2.2. FTIR analysis

**Fig 5:**
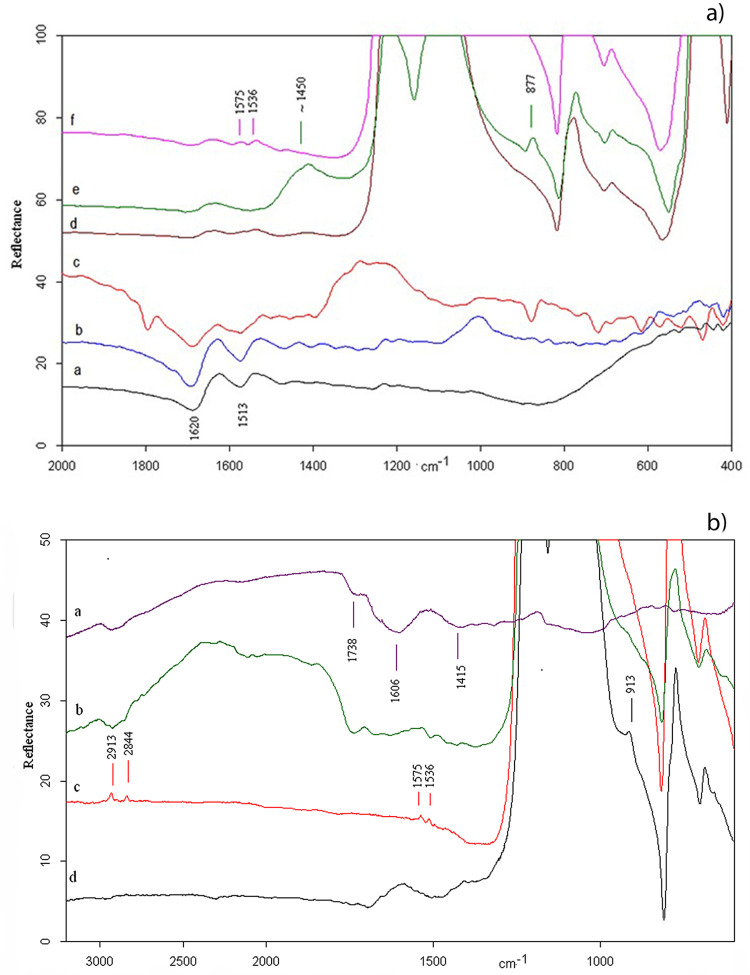
MicroFTIR of replicas: a) animal matters–(a) fresh hide, (b) dry hide, (c) hide and ash combined, (d) flint contact with hide, (e) flint contact with hide and ash combined, (f) flint contact with fresh hide some months later; b) plant matters–(a) inner side of asphodel, (b) flint contact with peeled asphodel, (c) flint contact with peeled asphodel some months later, (d) flint contact with unpeeled asphodel.

*3*.*2*.*2*.*1*. *The experimental replicas*. Active areas of replicas used in the processing animal or vegetal matters were analyzed using Fourier Transform InfraRed (FTIR) microspectroscopy. The spectra of both the used replicas and the worked matters are shown in Fig 5. Regardless of the processed material, the flint replicas show very intense absorption peaks at 1157 cm^−1^ assigned to Si-O stretching mode and less intense bands at 798 and 469 cm^−1^ respectively attributed to O-Si-O bending modes and to O-Si-O or O-Si-Al bending [77, 78]. All peaks show an updown reversal due to the Reststrahlen effect [79, 80].

The spectroscopic patterns of fresh and dry hide are presented in Fig 5a (*a* and *b*, respectively). In both cases, absorption peaks at 1620, 1513, 1445 and 1230 cm^-1^ are present and respectively assigned to C = O stretching (amide I), N-H bending and C-N stretching (amide II), C-H scissoring vibration of CH_2_, and CH_3_ groups, and amide III band of collagen, the most abundant protein in hide [81–85]. The dry hide spectrum (Fig 5a*b*) additionally shows a broad band at ~1000 cm^-1^ due to molecular changes induced by the drying process. However, as this absorption peak falls under the most intense band of flint, the infrared spectra were unable to distinguish between residues of fresh and dry hides remains on the flint tool [86].

Fig 5a and 5c shows the spectrum of hide preserved with ash, the principal component of which is calcium carbonate (CaCO_3_) [87]. As expected, a broad band at ~1450 cm^-1^ and two weak bands at 877 and 1794 cm^-1^ emerged respectively, assigned to the C-O stretching of CO_3_ the O-C-O bending, and a combination mode of CaCO_3_. The former peak partially overlaps the amide II and III bands of collagen. In fact, all amide bands are present in the spectrum of a flint replica used to work fresh hide (Fig 5a*d*), while only amide I band of collagen is present in the spectrum of a flint item used to work hide with ash (Fig 5a*e*).

The spectroscopic analysis of the flint tool with which fleshy tissues were worked (Fig 5a*d* and 5a*f*) was repeated twice: once when the tool had just been used and fleshy tissues were very fresh, and again after a few months, when the tissues had dried. In the second analysis spectra, a doublet at 1575 and 1536 cm^-1^, shows the transformation of the fresh fat tissues into adipocere upon drying due to the anaerobic bacterial hydrolysis of fat tissues [88], which is attributable to the C-O stretching mode of fatty acid salts (palmitate and/or stearate) [89–94]. Adipocere survives for extended periods of time because of its waxy, water-insoluble consistency which allows its penetration into the microholes of the flint stone.

Other flint replicas were used to work asphodel, which exhibits, as whole (or other) herbaceous plants, cellulose as a main residue accompanied with a minor amount of lignin [95] (Fig 5b).

Fig 5b shows the infrared spectra of the inner side of a fresh asphodel (Fig 5b*a*), of a flint replica used to peel fresh asphodel (Fig 5b*b*), of the same flint replica after few months (Fig 5b*c*), and of a flint replica that processed an unpeeled asphodel (Fig 5b*d*).

In accordance with previously reported cellulose spectra [95, 96], the infrared spectra of the inner side of a fresh asphodel (Fig 5b and 5a) show broad absorption peaks at 1738, 1606, and 1415 cm^-1^, respectively, attributed to C = O stretch, aromatic skeletal vibrations plus C = O stretch and CH_2_ scissoring. The flint replica used to peel fresh asphodel (Fig 5b*b*) shows the same spectroscopic bands. The same flint replica was analyzed a few months later under identical experimental conditions (Fig 5b*c*). This time, bands attributed to cellulose have disappeared leaving weak doublets at 2913/2844 and 1575/1536 cm^-1^ that suggests the existence of microresidues of the aforementioned acid salts [89, 93]. As shown in the spectrum, these salts are present in much lower amounts compared to those found on the flint replicas used to process fleshy tissues. This is because the lipidic components of the vegetal tissues are fewer than those present in fleshy tissues. On the flint tool used to work unpeeled asphodels (Fig 5bd), the most intense band emerges around 1600 cm^-1^ while the shoulder at ~913 cm^-1^ indicates the presence of the kaolin component (aluminum hydro silicate of formula Al_2_Si_2_O_5_(OH)_4_) originating in soil affixed onto the skin of the USOs itself [97].

*3*.*2*.*2*.*2*. *The Archaeological flint tools*. A few unwashed archaeological tool samples revealed a spectroscopic shoulder at ~913 cm^-1^ attesting to the presence of kaolin in areas related to the active edges of the tools or their prehension area. While kaolinite was indeed present in fireplace sediments of Qesem Cave [43], this residue may also be attributed to the P-O stretching mode of the PO_4_^3-^ group of hydroxyapatite (Ca_5_(PO_4_)_3_OH) [97–101], which represents the mineral component of bones. Thus, the spectroscopic analysis alone is insufficient to discern the nature of micro-residues without the help of use-wear or other independent residue analyses (see Tables 5 and 6; Figs 3e–3g and 4c and 4g).

**Fig 6:**
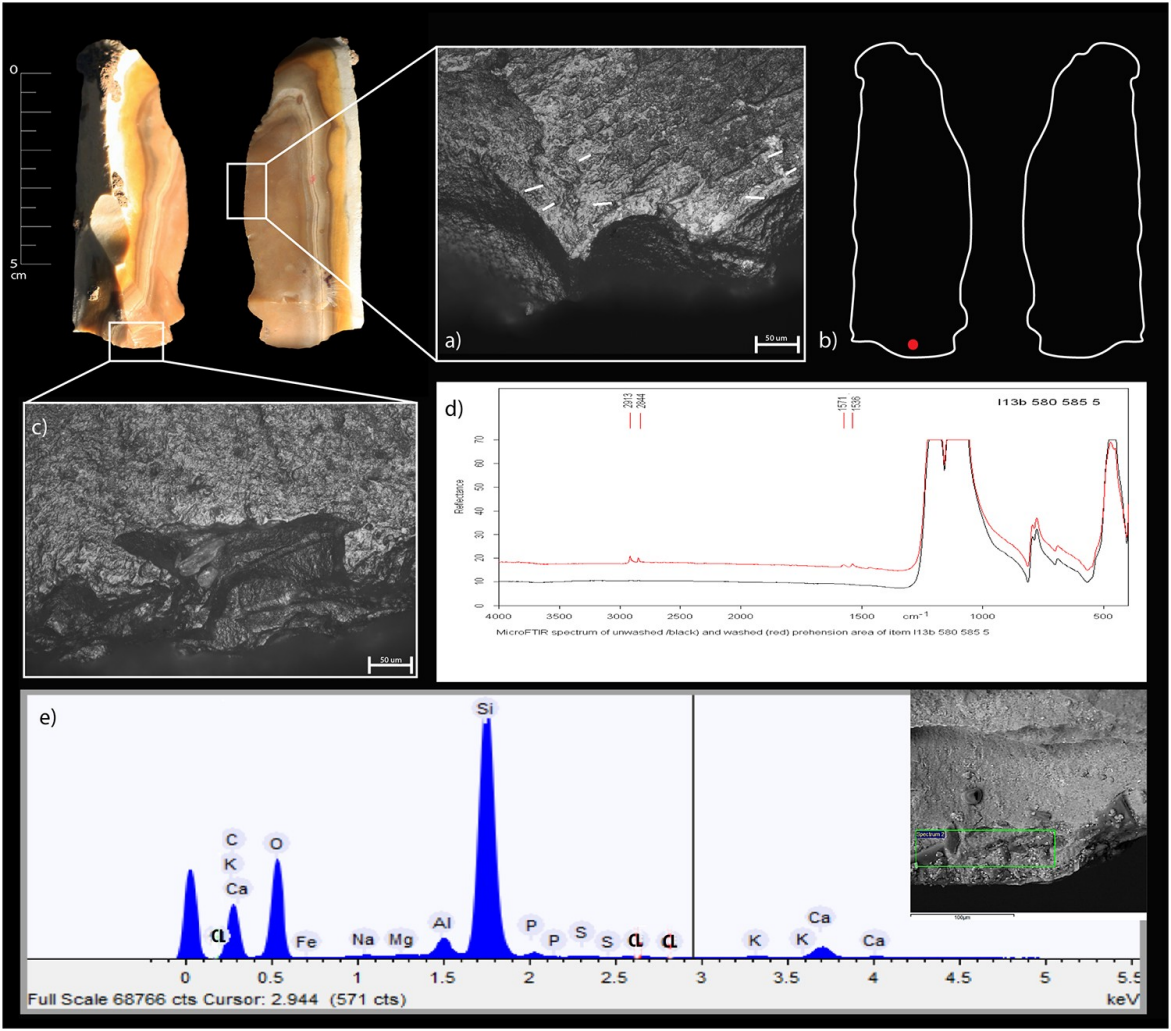
Qesem Cave, item I13b 580–585 5 a) use-wear of contact with ash-laden dry hide, b) item delineation showing the areas of FTIR analysis (in red), c) use-wear of free-hand prehension combined with ash; d) MicroFTIR spectrum of prehension area; e) SEM-EDX spectrum of active edge residues.

Unwashed archaeological tools also exhibit absorption peaks of ash [87]. Since the broad peacks of ash could have possibly covered peacks of other residues, these items were washed (see Methods above) and reanalyzed. Their post-wash analysis revealed the doublet at 1575 and 1536 cm^-1^, attesting to the presence of fatty acid salts in the active edge or prehension areas of the tool (Figs 4c, 4g and 6b and 6d).

The limited equivalency of finds in comparison data obtained from other archaeological and experimental tools suggests that collagen and cellulose are highly perishable substances, seldom traceable on archaeological artifacts. On such artefacts, it is more probable to find micro-residues of more resistant materials such as transformation products or mineral components.

#### 3.2.3. SEM-EDX analysis

**Fig 7:**
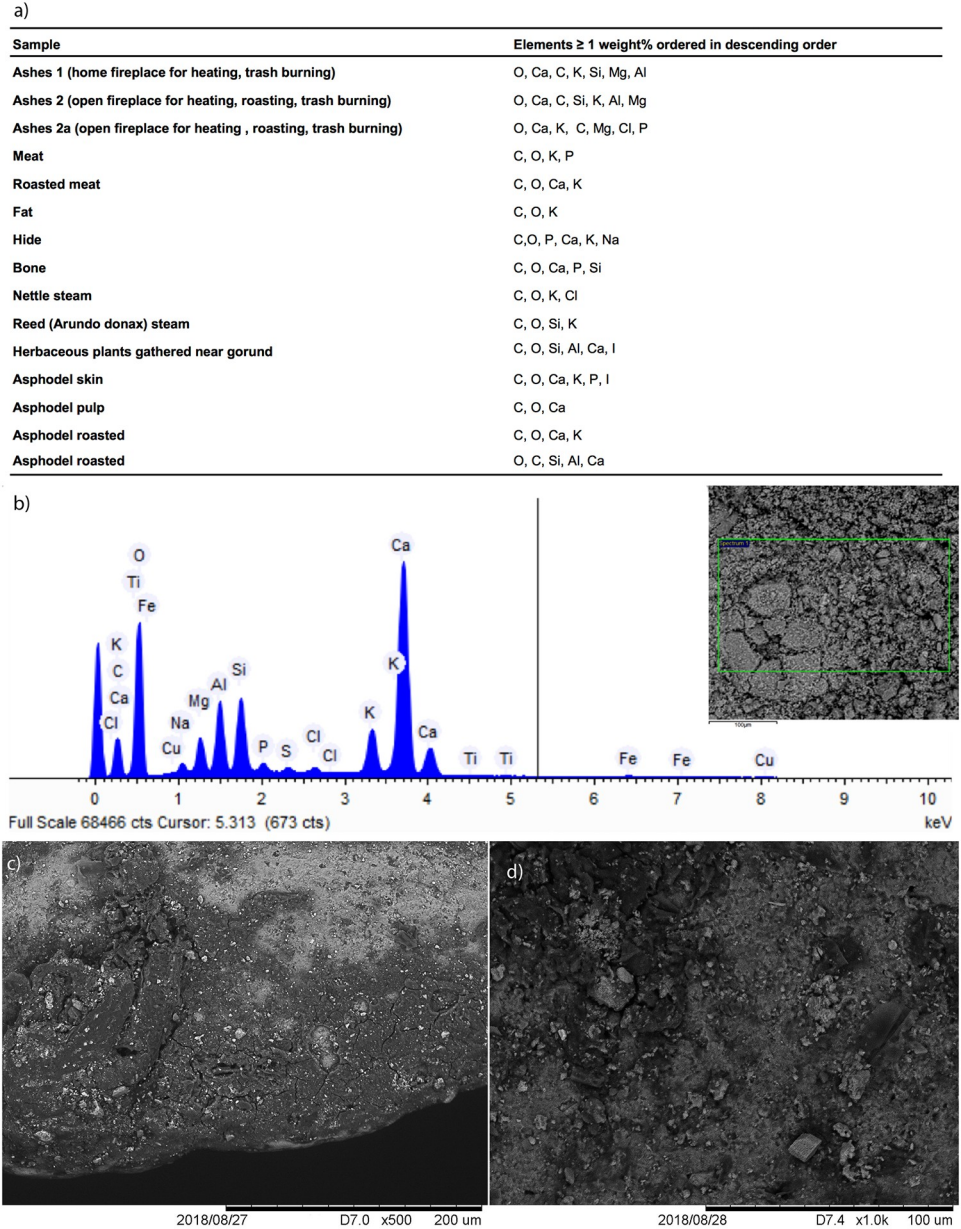
SEM-EDX: a) table of main chemical elements detectible on ash and on animal and plant matters, both raw and roasted, b) spectrum of ashes; SEM distribution and morphology of matters processed with ash on a blade replica–c) over its active edge, and d) over its prehension area.

*3*.*2*.*3*.*1*. *The experimental replica*. Analyzing the dedicated reference collection showed that SEM-EDX is advantageous in detecting inorganic mineral hydroxyapatite of bones [48, 57, 68]. Other than that, however, elemental analysis (EDX) does not seem to be advantageous in discriminating between plant and animal matters as the major components of both are oxygen (O) and carbon (C) whereas only minor components that vary according to the nutrients absorbed by animals and plants from their environment (see SI and Fig 7a). Notwithstanding, our EDX analysis confirmed, in accordance with our reference collection, the presence of ash and soil based on the detection of calcium, aluminum, and iron (Fig 7a and 7b).

Our SEM observation showed ash as a well-recognizable powder comprising grains of different sizes. In the reference collection, ash and other particles of processed animal or plant matters, formed a band of residues often entrapped on the use-scars of the active outer edge (Fig 7c). In the prehension area, these mixed residues formed small localized patches (Fig 7d).

*3*.*2*.*3*.*2*. *The archaeological flint tools*. The SEM-EDX combination allows for a comparison of the spectographic patterns of the archaeological artefacts with those produced on the flint replicas used for processing matters mixed with ash. In particular, the morpho-chemical patterns of ash are easily traced to their source and thus exhibit well-recognizable fingerprints (Tables 5 and 6; Figs 3f, 4e and 6e).

### 3.3. Use-wear analysis

#### 3.3.1. The experimental replicas

**Fig 8:**
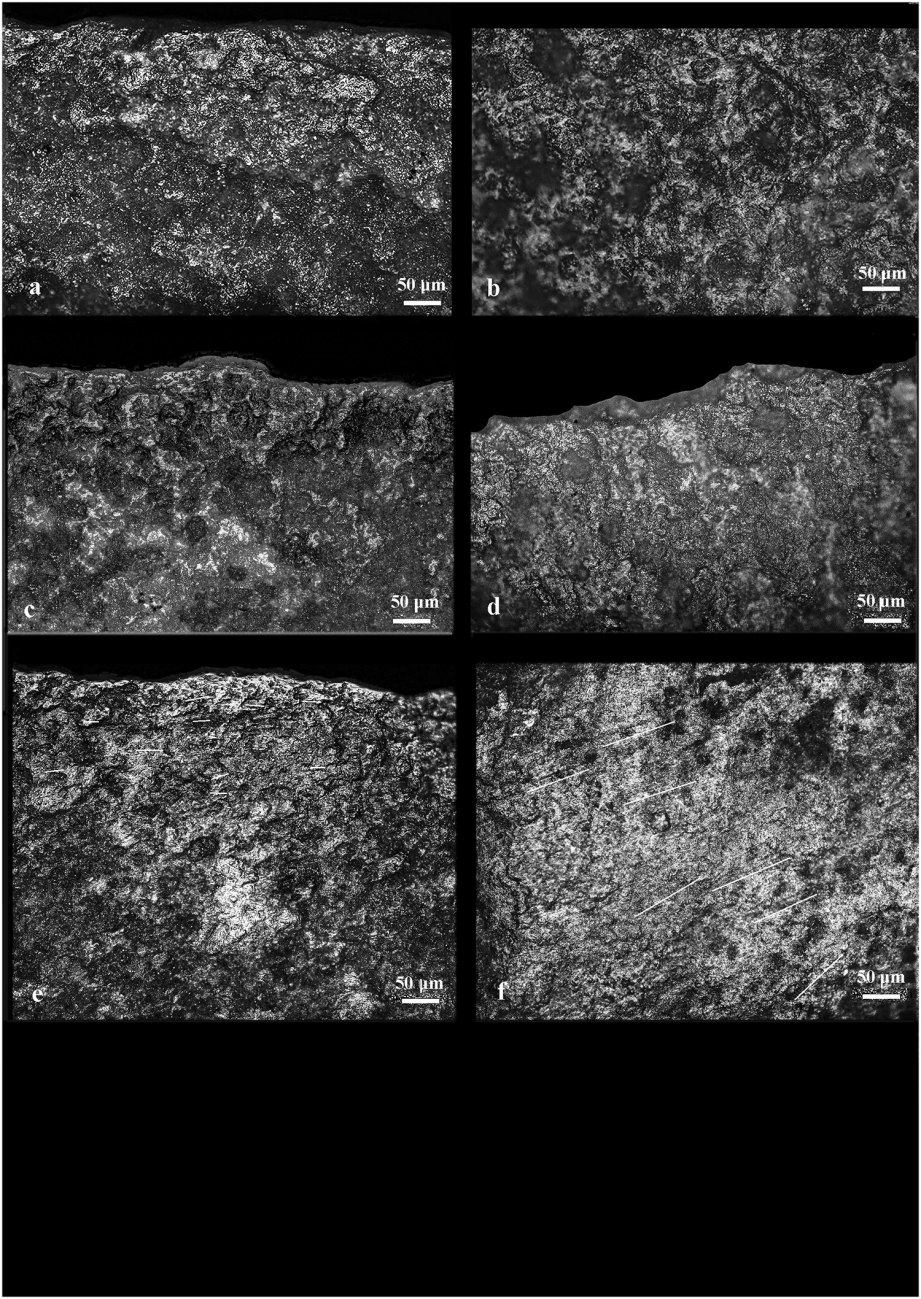
Use-wear of lithic replicas resulting from hide processing: ash-free fresh hide–a) the active area and b) the prehensive area; fresh hide contaminated by ash c) the active area and d) the prehensive area; dry hide preserved in ash–e) the active area and f) the prehensive area. Note the increase in linkage of polish in the active area of (e) as the polish extends into a band and while maintaining the characteristics of hide polish (smooth texture with a granular topography) it is more brilliant and reveals more developed edge rounding. The orientation of the small, thin striae (shown as white lines) in the active and prehensive areas reflects the direction of the processing movement.

**Fig 9:**
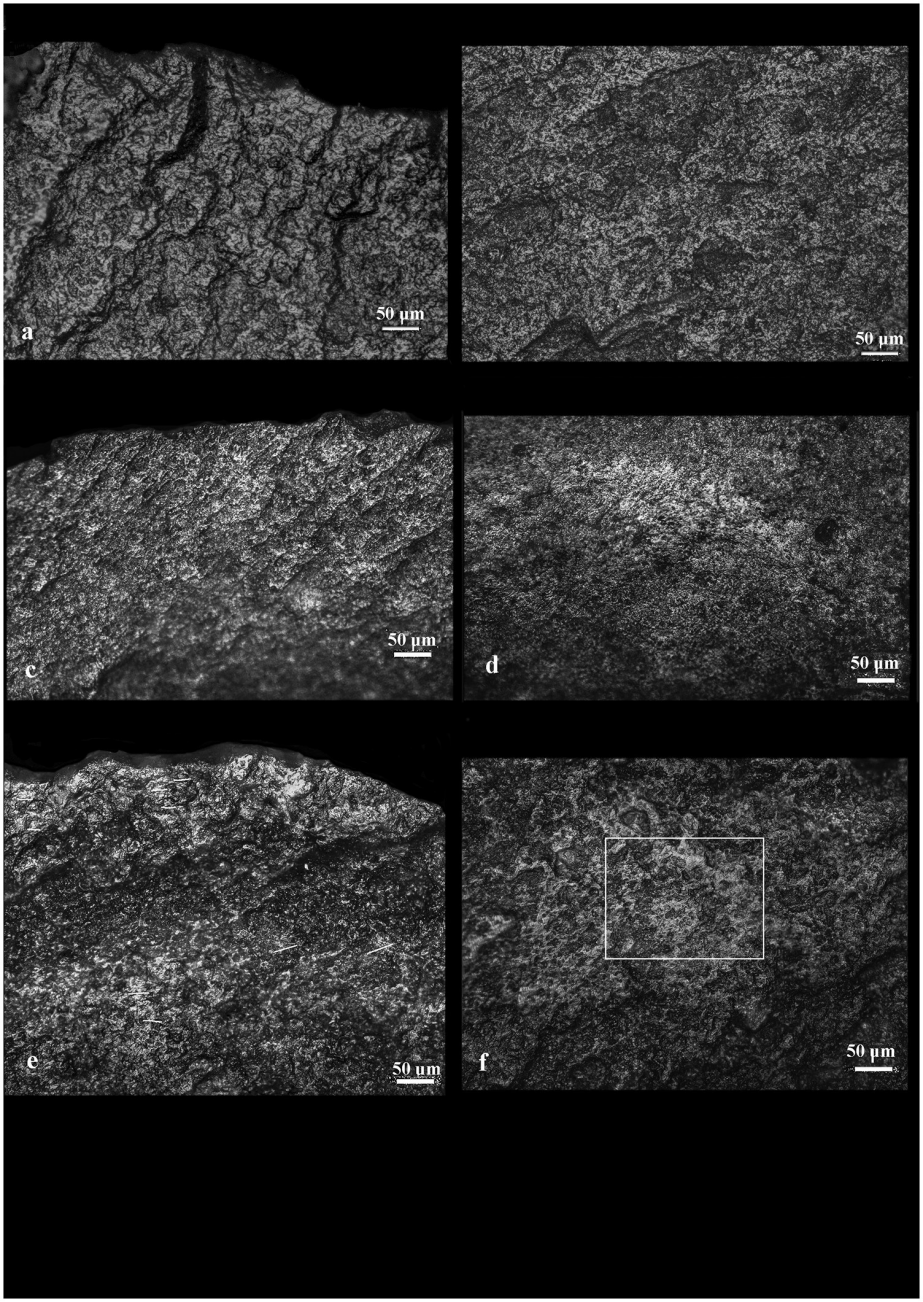
Use-wear of lithic replicas resulting from the processing of USOs: fresh USOs–a) the active area and b) the prehensive area; fresh USOs contaminated by ash–c) the active area and d) the prehensive area; roasted USOs–e) the active area and f) the prehensive area. Note the increase in linkage of polish in the active area (e) as the polish extends into a band and while maintaining the characters of the USO polish (rough to smooth texture with a granular or domed topography) it is more brilliant and reveals a more developed edge rounding. The orientation of very small and thin striae (shown as white lines in some cases) in the active and prehensive areas reflects the direction of the processing movement.

*3*.*3*.*1*.*1*. *Open analysis*. Comparing use-wear marks found on replica flint tools used to process ash-free (Figs 8a and 9a) and ash-laden (Figs 8e and 9e) matters revealed that use-wear of ash-free matters are highly localized on the active edge (outer edge or edge distribution) whereas the use of ash develops use-wear that appears as a band along the active edge.

This band-like distribution is uniquely related to the soft and abrasive consistency of the mixed ashy residues that facilitates the diffusion and development of the polish over a wider surface along the active edge. Although the peculiar use-wear traits of the worked matters (e.g., meat, herbaceous plants) [47, 72–74] often remain visible and distinguishable, ash enhances the rounding of the active edge, the smooth appearance of the polish in the high reliefs of the microsurface, and the polish linkage. In many cases, narrow and short striations due to the abrasiveness of the ash often appear on polished areas. Another specific feature of ash-laden matters is the development of matt and striated polishes due to the infiltration of the abrasive ash particles into the low areas of the flint micro-surface. Prehension areas (Figs 8b, 8f and 9b and 9f), generally localized on the distal and proximal ends as well as on the dorsal ridges of the flint tools, show well-developed polish spots (Figs 8f and 9f) characterized by the same smooth and bright appearance present on the active edges that is attributable to contact with ash. Often, thin striations are associated with polish are found on such areas.

*3*.*3*.*1*.*2*. *Blind test analysis*. The analysis of the flint replicas pertaining to the blind test was conducted by CL and FV under the assumption that wear features developed on the active edges of flint tools while processing plant and animal matters mixed with ash could be isolated and easily recognized. The results of the blind test proved this assumption (Table 4).

As noted earlier, three distinct patterns emerged in our analyses where active edges of the flint tools were used to process animal or plant matter mixed with ash (ash-laden): (a) active edges were noticeably rounded; (b) a bright, linked polish developed on the reliefs of the microsurface exhibiting minute, shallow striations; and (c) a striated matt polish was made visible in the low areas of the micro-surface (Figs 8e and 9e). These characteristics may be considered the fingerprints of the intentional use of ash as an additive as they were not identified on the blind test flint replicas utilized in proximity of an ash-rich environment or on tools contaminated by ash (such as through ashy hands). On such tools, use-wear found on active edges resembled that associated with the processing of the ash-free matter except for few small, ephemeral spots of bright and smooth polish scattered across the entire surface of the tool (Figs 8c and 9c). Such inconsistent and incoherently distributed spots of polish indicate that very few ash particles had accidentally migrated from the environment or the hands of handlers to the active edge of the tool from which they would have continuously been removed during the activity (Fig 2b and 2e; Repository Video 1).

Contrary to this, one result of the blind test showed that, while it was possible to identify the presence of ash related use-wear on the prehension areas of the tools, it was not possible to distinguish whether this use-wear pattern in the prehension areas referred to the processing of matters with the intentional addition of ash or to the manipulation of the tool by a user with hands contaminated with ash (Figs 8b, 8d, 8f and 9b, 9d and 9f).

#### 3.3.2. The archaeological flint tools

**Fig 10:**
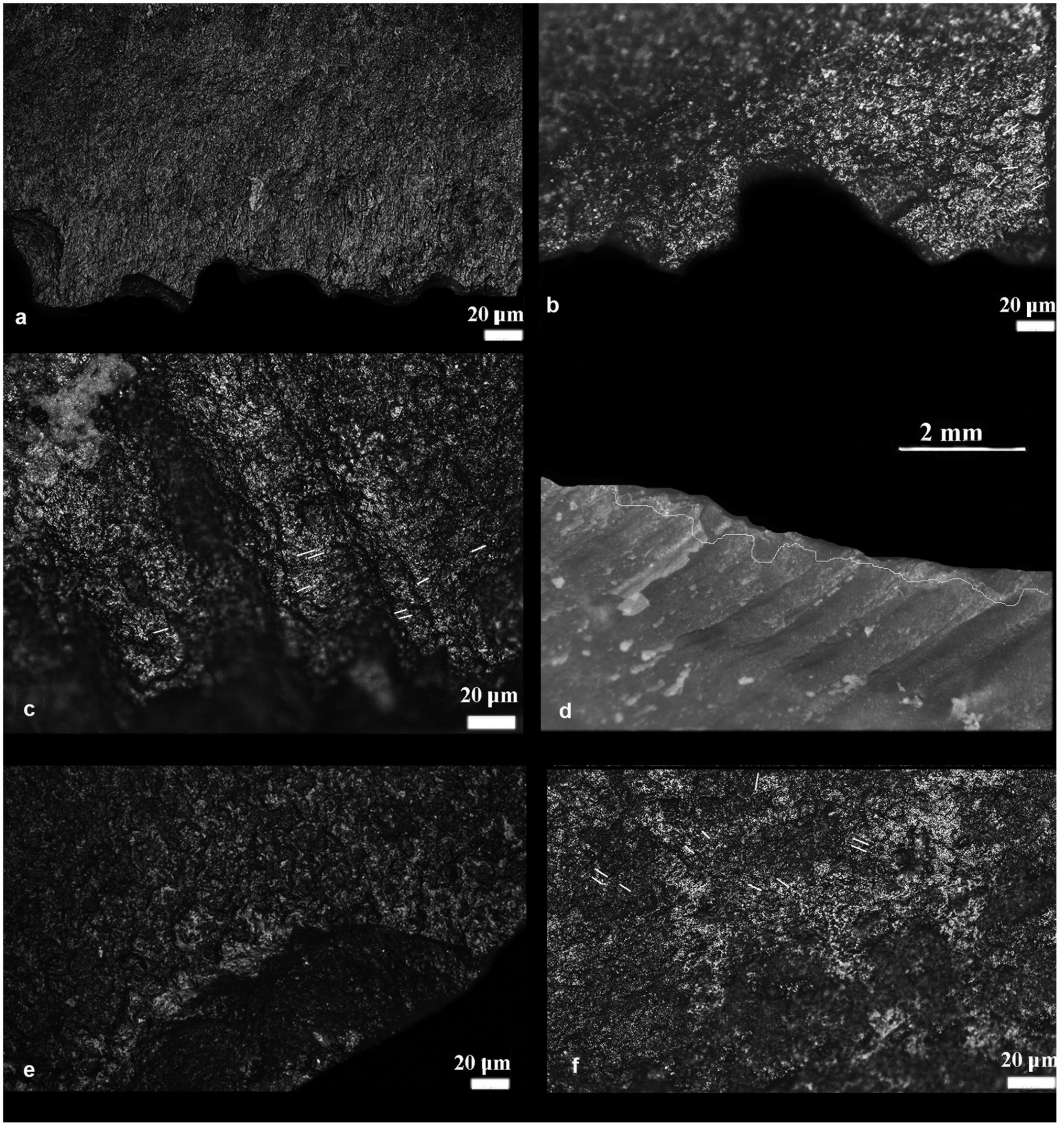
Examples of use-wear observed on lithic tools from the fireplace area of Qesem Caver: a) item I13d_570–575 1 active area, use-wear of contact with meat; notice the lack of the features (brightness, tight linkage and striae) typical of the contact with matters processed with ash; b) item I12 a 560–565 2 active area, use-wear of ash-laden fleshy tissues (band of polish, tight linkage, brightness, small thin striae); c) J13c 595–600 1 active area, use-wear of ash-laden USOs mixed (band of rough to smooth polish, tight linkage, brightness); d) item J13 c 595–600 1 active area, use-wear of medium material (edge-removals with an oblique orientation and a developed edge rounding); the orientation of the edge-removals suggests a longitudinal motion, cutting); e) item I13d_570–575 1 use-wear of free hand prehension; f) item I12 a 560–565 2 use-wear of free hand prehension plus ash particles (tight linkage and brightness, thin striae).

The 26 washed archaeological flint tools were examined under both low- and high-power approaches. The distribution and orientation of edge-removals proved to be particularly useful in reconstructing the motion used while employing the lithic tools (Fig 10d). Cutting was the primary activity carried out using these tools (*n* = 15), followed by tools used in activities involving both cutting and scraping (*n* = 5). A single tool exhibited features from which we were unable to characterize the motion that carried out with it (Table 5).

The comparison between the morphological features and patterns of the polish and striations that were identified on the archaeological tools and those identified on the experimental replicas allowed us to confidently infer the use of the archaeological tools in processing ash-laden fleshy tissues (*n* = 1) (Fig 10b), dry hide (Fig 6a) (*n* = 7), USOs (*n* = 6) (Figs 3c and 10c), and herbaceous plants (*n* = 4) (Fig 4a) (Table 7).

**Table 7:** Use-wear evidence of ash-laden processing on Qesem Cave lithic items from various cave areas.

Analyzed tools and originating area in cave	Ash-laden material traced on active edge	Item count per action				
		Cutting	Scraping	Engraving	Mixed	Total
Blades from Fireplace	Fleshy tissues	1				1
	Dry hide	3	1			4
	Non-woody plant	3			1	4
	USOs	2			4	6
	Soft material	1				1
	Soft to medium material	2	1			3
	Medium material		1			1
	*Total*	*12*	*3*		*5*	*20*
Recycled small flakes from Fireplace	Soft to medium material				1	1
Recycled small flakes from the Area South of the fireplace	Soft to medium material	2				2
Quina scrapers from Shelf	Dry hide		3			3

In the remaining cases, traces related to the use of ash were clear whereas the nature of the processed ash-laden matter was not, other than its soft or medium consistency revealed through edge removal patterns and the degree of edge rounding affecting the active edge of the tools.

Polish and striations patterns developed on the prehensive area of the experimental replicas share the characteristics identified on the archaeological specimens, suggesting that the latter were used mostly hand-held. Some features seem to suggest that ash particles were present on handlers’ hands; these include the smooth bright striated polish found on the prehensive area (Table 6; Figs 3d, 4b, 6c and 10f).

For the sake of comparison, we present in Fig 10 also examples of use-wear preserved on the active edge and prehension area of lithic tools found in the fireplace area of Qesem Cave that were involved in the processing of both ash-free (Fig 10a and 10e) and ash-laden matters (Fig 10b–10d and 10f).

### 3.4. Matching residues and use-wear

#### 3.4.1. Active edge

Combining findings from our residue and use-wear analyses further supports our interpretations regarding the intentional use of ash while processing animal or plant matters (Table 2). Thus, when present, the morphological characteristics of the residues match perfectly with the inference indicated by the use-wear analysis. Moreover, residue analysis may indicate the type of matter mixed with ash in cases such as item I13a 560–565 1, where the inference obtained through use-wear analysis allowed to precisely determine the activity carried out (cutting), and less precisely the (soft) matter that was combined with ash. The fat acid salt, detected through our FTIR analysis, which had originated in the degradation of fatty animal or plant tissues confirmed the findings of the use-wear analysis in three active edges (particularly relating to USOs). The presence of organic matter mixed with ashes and soil, established in our SEM-EDX analysis through the occurrence of O, C, Ca, Al, and I, matches use-wear patterns detected on the active edges of five items. Similarly, plant fibers corresponding with use-wear indicate the processing of USOs with ash (I13b 590–595 5). However, collagen fibers observed on the same item suggest some overlap in activities involving materials that were not expressed in the use-wear analysis or some migration of contamination from the sediments.

#### 3.4.2. Prehension areas

Fat acid salt residues found through FTIR analysis on five items out of eight indicates the migration of particles of organic matters to the prehension area during work. Our use-wear analysis supplements this finding, further suggesting that in four cases (Table 3), the tools were handled by hands dirty with ash particles. In addition to ash, we further found through SEMEDX analysis chemical elements coherent with organic materials and soil in one of these five items (I13d 590–595 1).

## 4. Discussion and conclusions

Ash is clearly generated at Qesem Cave in large quantities [31] throughout the sequence of the cave. Retrospectively, then, it should have been regarded as a research target.

A brief survey of the ethnographic record shows a variety of activities in which ash had been utilized. These may be divided into four major categories: a) food roasting and cooking [24]; b) preservation of edible matters, such as raw seeds [23], raw USOs [20, 26], dried food [26], or other matters such as fresh hide; c) hygiene treatments of dwellings aimed at keeping insects and parasites at bay or the prevention of insect proliferation [25], and d) medicinal uses [24].

With respect to the second use mentioned here, mixtures of ash and soil are utilized by communities worldwide for long-lasting food preservation [27] as cold and hot ashes inhibit organic decomposition and the rotting odor of organic matters. Traditional conservation techniques include roasting, followed by the covering of the product with ash or a mixture of ash and soil [28]. In traditional tanning procedures, ash is comparable to salt in terms of preservation efficiency of fresh hide [20].

The analytical methods presented in this study did not allow us to detect any evidence for the use of ash in hygiene purposes or medicinal use [but see 102–104].

Our own study focused on evidence concerning the roasting and preservation of food and other matters (e.g., hide). Our multi-perspective study of ash uses at 420–200 kya site of Qesem Cave aimed at revealing patterns that could be uniquely associated with the intentional use of ashes in the processing of organic matter. The identification of these activities was also made possible due to the highly preserved flint tools, where residues could be recognized as well as use-wear signs on the tools. Indeed, the combination of the methods applied in this study allowed us, for the first time, to uncover a specific fingerprint of polishes and striations directly related to the use of ash, supported by congruent residues.

Accompanied by a dedicated experimental protocol designed to verify use-wear characteristics of different matters processed with or without ash, the results of our use-wear analysis of the archaeological tools was also compared with the rich reference collection of the LTFAPA laboratory. This analysis demonstrated ash-laden USOs and dry hide were processed at Qesem Cave especially around the fireplace (Table 7). Our study unequivocally links the use of ash to the roasting and storing of USOs as well as to hide preservation. Evidence emanating from the processing of ash-laden herbaceous plants, however, cannot be clearly interpreted as being related to foods. The presence of an item exhibiting processing traces of ash-laden fleshy tissues supports the notion that ash at Qesem Cave was additionally used in animal food processing.

The polish and striations patterns identified on 20 flint blades and 6 additional flint tools suggest that at this site, and especially around its large central fireplace, hominins roasted plant and animal foods and stored food and other matters, such as hide, while taking advantage of the preservation properties of ash as early as ca. 300 kya. Food preservation is an important aspect in our understanding of human behavioral evolution. The evidence from Qesem Cave presented here is well in accordance with the results of a recent study on fallow deer metapodia, which seems to attest to the long-term (weeks or even months) storage of marrow [105].

The study presented here demonstrates the power of a multi-perspective investigation of prehistoric flint tools in detecting the use of specific actions over specific types of matters. Specifically, our combination of use-wear and residue analyses compared to a dedicated reference collection of experiments and further supported by a blind test allowed us to reveal the hallmark on flint tools related to the processing of animal and plant matters intentionally mixed with ash for (delayed) consumption or processing purposes. These use-wear fingerprints comprise a band along the active edge comprising smooth bright striated polish developed on micro-surface reliefs combined with matt striated polish developed in the low areas of the flint micro-surface. In contrast, experimental flint tools used in a context in which ash was present in the working environment and not intentionally used for the purpose of the activities in which the flint tools were employed, show only small spots of smooth bright and striated polish spread on the micro-surface of the tool.

Within this greater picture, use-wear analysis was found useful in inferring the intentional use of ashes through the analysis of active edges. It was not, however, useful for assigning such use to particular activities such as roasting or storing as use-wear signs where ashes were present (e.g., of USO processing) could be interpreted both ways. Unequivocal interpretation of preservation purposes was limited to processed matters other than food, such as hide. The analysis of use-wear traces on prehensive areas did not contribute to the inference of intentional versus unintentional presence of ash, and we were unable to distinguish between prehension traces related to the processing of matters mixed with ash from traces generated by accidental contamination (such as by ashy hands).

Residues analysis was also useful in providing information concerning the processed substances but not concerning the intentional or unintentional use of ash as an additive. Through our employment of different analytical techniques (e.g., morphological analysis of residues, FTIR, SEM-EDX), we were able to support and confirm the results of the use-wear analysis. Nevertheless, the intentional use of ash became discernable only through the identification of specific use-wear patterns that were supported by residue analysis.

We thus confirm that the particular polish and striations patterns (fingerprints) observed on the archaeological items from Qesem Cave coupled with the evidence of preserved residues shed light on activities otherwise invisible, opening a window to the possibility of further detection of specific behaviors at the site (or elsewhere). The presence of the microscopic use-wear fingerprints found on Middle Pleistocene flint tools at Qesem Cave introduces into the debate on fire, pyro-technology, and secondary derivatives (by-products) of fire the unexplored benefits of the use of ash for human livelihood and human evolution. Our results indicate that USOs and other plants were probably roasted or preserved (in ash) for delayed consumption, and that ash appears to have been used for treating and preserving raw hide. This suggests that the site’s inhabitants had already mastered the outstanding properties of ash for roasting/cooking and preservation purposes [25, 28]. These results accord well with data concerning faunal remains found in the hearth area of the cave, which reveal recurrent roasting of meat and the manipulation of bones for marrow extraction and delayed consumption [10, 105, 106]. Our results are also in line with the direct evidence found at the site for human exposure to an ashy environment, revealed through the recovery of micro-charcoal particles in the dental calculus of three hominin teeth found at Qesem Cave [107]. The data and findings of the current study complement previous findings at this site, further exposing the skills of the Late Lower Paleolithic population at Qesem Cave to manipulate food and delay its consumption.

Our direct first-hand experience and controlled experimental work have corroborated the usefulness of ash in the preservation of dry untanned hides for months. Ash is also suitable for drying tendons that can be turned into strings or for drying fresh bone to ease the removal of fleshy tissues when manufacturing bone tools. Hominins’ use of ash may have also been related to improved hygiene, especially with respect of long-term (semi-) permanent base camp sites or closed environments such as rock shelters and caves, which are prone to housing harmful bacteria. Moreover, the use of ash to mitigate the odor of decomposing organic remains might have also reduced the attention of predators to hominin sites. The surprising lack of carnivore remains in the rich faunal assemblage of Qesem Cave [106] may be considered as supporting evidence for such an interpretation.

As such, Qesem Cave seems to provide the earliest evidence related to the utilization of ash for storing and processing vegetal foods and hide linked to the outstanding preservation properties of ash. If so, the evidence produced in this paper suggests quite an early timeline for these behavioral patterns, which to date have only been associated with much later (Upper Paleolithic, post 45,000 kya) communities in the Levant [108] and in Europe [11]. The use of ash for processing, preserving, and storing food and other matters (e.g., hide) as early as 300 kya at the site of Qesem Cave may be perceived as part of a new mode of adaptation characterizing the post-*Homo erectus* hominins of the AYCC [109]. Our results highlight the possibility that fire was used purposefully and indirectly (as opposed to its direct uses of heat, light, and security) in the Levant from ca. 300 kya onwards through the utilization of its by-product, ash.

In behavioral terms, the use of ash allowed for new options in the planning of daily activities through the storage of what might otherwise have been highly perishable foods. This would have had a significant impact on community adaptation strategies in times of food scarcity while also affecting mobility strategies, increasing the feasibility of more stationary occupations [109]. Our results call for a reassessment of the role of fire, fireplaces, and their by-products as indicators and promotors of a significant behavioral transformation some 300 kya at Qesem Cave as part of a broader cultural and evolutionary transformation that occurred in the Levantine Late Lower Paleolithic.

We have evidenced some aspects of this transformation in a plethora of technological innovations, such as blade production, a shift towards a diet comprising cooked and roasted foods originating in medium-sized ungulates, the adoption of new hunting techniques and butchering practices, delayed consumption of marrow, changes in mobility patterns and the appearance of more permanent occupation sites, and seemingly enhanced mechanisms for knowledge transmission [110]. Evidence unearthed at Qesem Cave suggests that these cultural elements were practiced by a newly evolved human lineage [37, 111] that appeared in the Levant ~400 kya. The use of ash, evidenced in the current study, is yet another aspect of this transformative evolution.

## Supporting information

S1 Fig Examples of use-wear from the South Area of the fireplace and from the Shelf testifying the processing of ash-free matters. South Area of the fireplace, recycled small flake item J15a 590–595, a) edge-removals and b) polishes of herbaceous plant; Shelf Area, Quina scrapers c) item **D7b 1085–1090**, polishes of bone general working, d) item E12b_560–580, polishes of hide scraping, e) item **G8a 630–635 polishes of wood cutting,** f) **G8a 625**, polishes of wood scraping.
(TIF)

S2 Fig Processing of roasted USOs of *Asphodelus ramosus* L. with replicas of blades. a- b) distribution of residues after experimental processing of USOs. Patches of sediment, plant and ash residues can be observed in some areas along the edge (a) although generally concentrated away from it (b). Spots of residues also show a patchy distribution; c) compressed appearance of the residues in the prehension area.
(TIF)

S3 Fig Type of residues associated to experimental processing of fibrous USOs plants. a) compressed appearance of residues left on the prehensile area of experimental tool used for cleaning USOs; b) Fibres with a patchy and compressed appearance distributed away from the edge and in the prehensile areas; c) Plant, soil particles and organic film showing an invasive distribution along the edge; d) Sticky and partly mudcracked organic film; e) Patched of residues distributed along and away from the edge; f) Close up on the fibres and raphids left away from the edge of a tool used for cutting USOs.
(TIF)

S1 Video (MP4)

S1 File (DOCX)
