# Supplementary figures and images for: The use of ash at Late Lower Paleolithic Qesem Cave, Israel—An integrated study of use-wear and residue analysis
Source: PLoS One. 2020 Sep 21;15(9):e0237502. doi: 10.1371/journal.pone.0237502 (PMC7505473; doi:10.1371/journal.pone.0237502)

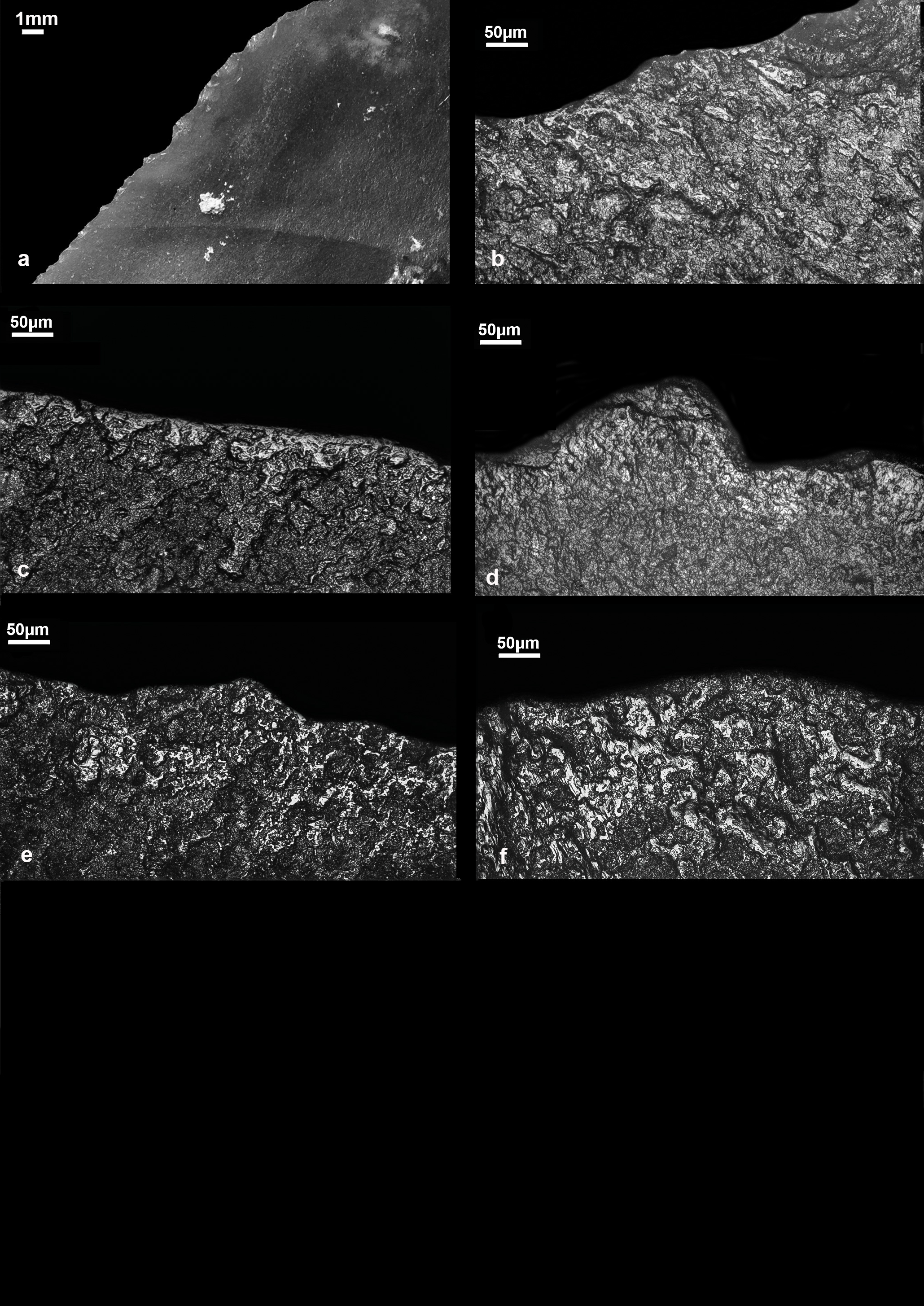

Supplement: S1 Fig — South Area of the fireplace, recycled small flake item J15a 590–595, a) edge-removals and b) polishes of herbaceous plant; Shelf Area, Quina scrapers c) item D7b 1085–1090, polishes of bone general working, d) item E12b_560–580, polishes of hide scraping, e) item G8a 630–635 polishes of wood cutting, f) G8a 625, polishes of wood scraping. (TIF) [file pone.0237502.s001.tif]

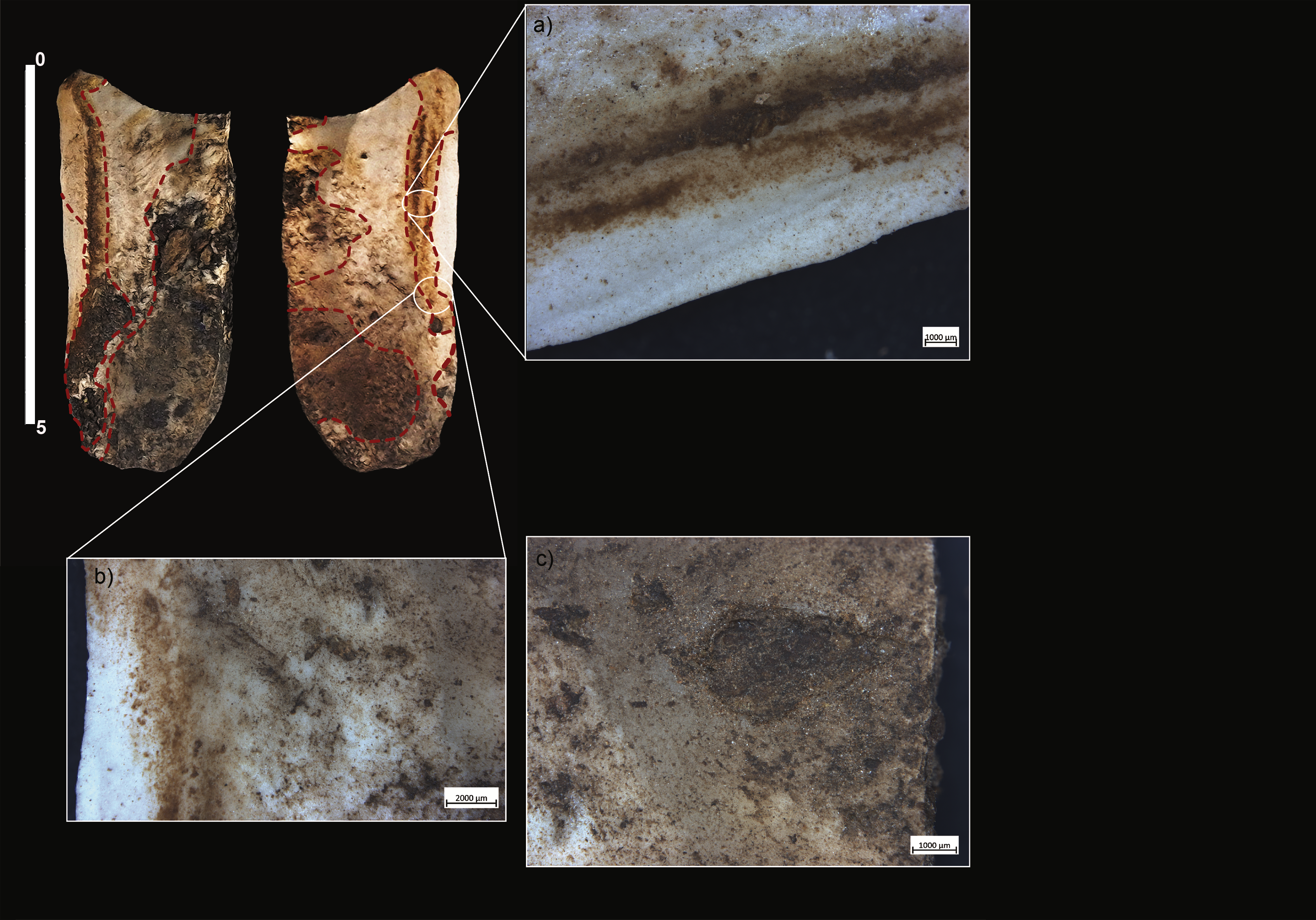

Supplement: S2 Fig — a- b) distribution of residues after experimental processing of USOs. Patches of sediment, plant and ash residues can be observed in some areas along the edge (a) although generally concentrated away from it (b). Spots of residues also show a patchy distribution; c) compressed appearance of the residues in the prehension area. (TIF) [file pone.0237502.s002.tif]
