# Supplementary material for: The use of ash at Late Lower Paleolithic Qesem Cave, Israel—An integrated study of use-wear and residue analysis
Source: PLoS One. 2020 Sep 21;15(9):e0237502. doi: 10.1371/journal.pone.0237502 (PMC7505473; doi:10.1371/journal.pone.0237502)
Supplement: S1 File — (DOCX) [file pone.0237502.s005.docx]

**Supporting Information**

**1. Use-Wear Analysis**

Low- and High-Power approaches need distinct Optical Light Microscope OLM systems and different magnifications to observe the related traces. In our work, a Nikon SMZ stereomicroscope capable of magnification up to 7.5x and a Nikon Elite Metallurgical microscope capable of magnification up to 500x were used for Low and High-Power approach. A digital camera ToupView and related software were used for the documentation of the use-wear on replicas. Polishes observed on the archaeological items were documented with a metallographic Zeiss Axio Scope A1 with magnification up to 400x and a digital camera Zeiss Axiocam 506 Colour. The focus of images has been processed using Helicon Focus software.

**2. Residues Analysis**

Residues have been observed in situ using a stereoscope Zeiss Axio Zoom (magnification ranging from 10x to 178x) as well as under a metallographic microscope Zeiss Axio Scope A1 with magnification ranging from 10x to 400x and a digital camera Zeiss Axiocam 506 Colour.

The focus of images has been processed using Helicon Focus software.

**3. FTIR Analysis**

The Fourier Transform InfraRed (FTIR) spectra were collected with a Bruker Optic Alpha-R portable interferometer with an external reflectance head covering a circular area of about 5 mm in diameter. The analysis does not require a preliminary treatment of the samples. The investigated spectral range was 7500–375 cm^-1^ with a resolution of 4 cm^-1^ cumulating 250 scans or more. The spectra reported in the main text however, show only the range where absorption peaks are observed. In most cases, the samples were analysed before and after washing. The internal point and edges of the replicas and archaeological tools were examined in the same experimental conditions.

The infrared spectroscopic analysis was conducted on replicas created by working animal or vegetal matters with flint items in an attempt to reproduce the observed archaeological activities.

Recently, an alternative method of spreading a homogeneous slurry of the possible worked materials on flint items has been proposed [112]. In this way obtained experimental tools, however, do not mimic the distribution of residues of the worked matter over the lithic surface.

**4.** **SEM EDX Analysis**

SEM-EDX analysis was carried out with a SEM Hitachi Tabletop TM3000 plus, an EDX system SwiftED3000 and related software allowing semi-quantitative analyses. The archaeological samples were analysed in total vacuum without coating. The EDX analysis was carried out at 15kVaccelerating voltage (BSE mode) with an acquisition time of 400 s.

For the sake of a comparison, a reference collection of EDX spectra of matters analogues to the matters possibly processed with the archaeological items was created. Every sample was mounted on aluminium stabs completely covered with carbon film on which the sampled matter was firmly stuck. Every sample was left to dry out at room temperature to avoid possible dispersion of water vapour during analysis inside the vacuum chamber. To verify the occurrence of a “fingerprint” combination of major chemical elements of each matter, we decided to take in consideration the chemical elements equal or major to 1.000 in weight %.

The comparative table is presented in Figure 7a of the main text. Depending on the context of production and fireplace use, ashes, whose major component is calcium (Ca), may vary in its minor chemical contents; they can vary in the same fireplace as well. Nevertheless, oxygen O (>50%) and calcium Ca (>20%) are always the main elements, followed by carbon C; potassium K can be also present in high percentages (Supplementary Figure S3b). When kaolinite particles from soil (see also MicroFTIR analysis above) are mixed with ashes, silicon Si and aluminum Al may also be detected. Soft animal tissues, such as meat, fat, hides, are characterized by C, O and K, and possibly other elements. Bone is characterized by a peculiar doublet between the elements calcium and phosphorus which is always detected in high percentages and in the fixed proportion of CA/P=1.6 [113]. Plant matter, aside from C and O, may present different combinations of elements depending on the part of the plant analyzed. As an example, the hard steams of *Arundo donax* are rich in silica structures (Si), the phytolites. The USOs’ skin absorbs from the ground various elements that are not present in its pulp.

Summing up our results, only few pure matters may be unambiguously detected with EDX chemical technique. Moreover, when animal and plant matters are processed with ashes, Ca may appear in matters where it is not present when plain (see the difference between the chemical elements composing row meat and meat roasted in Figure 7a of the main text) or Si and Al may appear when ash is mixed with ground particles (see the difference between the chemical elements composing asphodel fresh and roasted in Figure 7a of the main text). This brief discussion clearly demonstrates that plant or animal matters cannot be unambiguously interpreted with this technique when they are mixed with ash. Only a combination of EDX with SEM detection of residues with discernible morphologies may allow for a reasonable interpretation [57, 68].

| **The Hearth Area** | **no.** | **Sample Analysed with Use-Wear Approach** | **Items with Use-Wear** |
| --- | --- | --- | --- |
| Blade | 167 | 167 | 47 |
| Recycled small flakes | 297 | 156 | 46 |
| Quina scraper | 6 | 6 | 3 |
| **South of the hearth area** | **no.** | **Sample Analysed with Use-Wear Approach** | **Items with Use-Wear** |
| Recycled small flakes | 230 | 43 | 12 |
| **Square K10** | **no.** | **Sample Analysed with Use-Wear Approach** | **Items with Use-Wear** |
| Blade | 495 | 253 | 74 |
| **Shelf** | **no.** | **Sample Analysed with Use-Wear Approach** | **Items with Use-Wear** |
| Quina scraper | 119 | 119 | 54 |

**SI Table 1**

| **Blades Fireplace Items 27** | **Material/Action**  **(Active Edge)** | **Cutting** | **Engraving** | **Mixed** | **Scraping** | **Total** |  |
| --- | --- | --- | --- | --- | --- | --- | --- |
|  | Fleshy tissues | 4 |  |  |  | **4** |  |
|  | Fresh Hide + Meat | 1 |  | 1 |  | **2** |  |
|  | Fresh Soft Wood | 1 |  |  |  | **1** |  |
|  | Non-woody plant | 2 |  |  | 2 | **4** |  |
|  | USOs | 1 |  |  |  | **1** |  |
|  | Soft Material | 8 |  |  |  | **8** |  |
|  | Soft to Medium Material | 3 |  |  |  | **3** |  |
|  | Medium Material | 1 | 1 | 1 | 3 | **6** |  |
|  | **Total** | **21** | **1** | **2** | **5** | **29** |  |
| **Recycled small flakes Fireplace Items 45** | **Material/Action**  **(Active Edge)** | **Cutting** | **Mixed** | **Scraping** | **Total** |  |  |
|  | Fleshy tissues | 12 | 4 |  | **16** |  |  |
|  | USOs |  | 1 |  | **1** |  |  |
|  | Soft Material | 17 | 1 | 1 | **19** |  |  |
|  | Medium Material |  | 1 |  | **1** |  |  |
|  | Soft to Medium Material | 5 | 3 |  | **8** |  |  |
|  | **Total** | **34** | **10** | **1** | **45** |  |  |
| **Quina scrapers Fireplace Items 6** | **Material/Action**  **(Active Edge)** | **Cutting** | **Mixed** | **Total** |  |  |  |
|  | Flesy tissues | 2 | 1 | **3** |  |  |  |
|  | Dry Hide | 1 |  | **1** |  |  |  |
|  | Soft material | 2 |  | **2** |  |  |  |
|  | **Total** | **5** | **1** | **6** |  |  |  |
| **Recycled small flakes Area South of the fireplace** | **Material/Action**  **(Active Edge)** | **Cutting** | **Total** |  |  |  |  |
| **Items 10** | Fleshy tissues | 6 | **6** |  |  |  |  |
|  | Bone | 1 | **1** |  |  |  |  |
|  | Plants | 2 | **2** |  |  |  |  |
|  | Soft to Medium Material | 1 | **1** |  |  |  |  |
|  | **Total** | **10** | **10** |  |  |  |  |
| **Blades Square K10 Items 74** | **Material/Action**  **(Active Edge)** | **Cutting** | **Engraving** | **Mixed** | **Scraping** | **Whittling** | **Total** |
|  | Fleshy tissues | 13 |  | 1 | 3 |  | **17** |
|  | Fleshy tissues + Bone | 1 |  | 1 | 2 |  | **4** |
|  | Hide | 2 |  |  | 3 |  | **5** |
|  | Wood/Plants | 3 |  | 2 |  |  | **5** |
|  | Medium Material | 1 |  | 1 | 8 |  | **10** |
|  | Soft to Medium Material | 6 | 1 | 3 | 1 |  | **11** |
|  | Soft | 25 | 1 | 2 | 1 | 2 | **31** |
|  | Indeterminable | 3 |  |  | 2 |  | **5** |
|  | **Total** | **54** | **2** | **10** | **20** | **2** | **88** |
| **Quina/Demi-Quina scrapers Shelf Items 51** | **Material/Action**  **(Active Edge)** | **Cutting** | **Mixed** | **Scraping** | **Total** |  |  |
|  | Fleshy tissues | 1 |  | 1 | **2** |  |  |
|  | Bone | 3 | 1 | 2 | **6** |  |  |
|  | Hide | 1 | 1 | 22 | **24** |  |  |
|  | Wood | 2 | 3 | 11 | **16** |  |  |
|  | Plants | 1 | 1 | 2 | **4** |  |  |
|  | Medium Material |  | 2 | 2 | **4** |  |  |
|  | Soft to Medium Material | 3 | 4 | 1 | **8** |  |  |
|  | Soft | 1 | 3 | 1 | **5** |  |  |
|  | **Total** | **12** | **15** | **42** | **69** |  |  |

**SI Table 2**

**SI Fig 1**

**SI Fig 2**

**SI Fig 3**

**References**

112 Monnier G. Developing FTIR microspectroscopy for analysis of plant residues on stone tools. J Archaeol Sci. 2017; 78: 158-178.<https://doi.org/10.1016/j.jas.2016.12.004>

113 Tariq, U., Haider, Z., Chaudhary, K., Hussain, R., Ali, J., 2018. Calcium to phosphate ratio measurements in calcium phosphates using LIBS. J. Phys. Conf. Ser. 2018 1027, 012015<https://doi.org/10.1088/1742-6596/1027/1/012015>
